# The Renaissance of Wild Food Plants: Insights from Tuscany (Italy)

**DOI:** 10.3390/foods11030300

**Published:** 2022-01-23

**Authors:** Ada Baldi, Piero Bruschi, Stephanie Campeggi, Teresa Egea, Diego Rivera, Concepción Obón, Anna Lenzi

**Affiliations:** 1 Dipartimento di Scienze e Tecnologie Agrarie, Alimentari Ambientali e Forestali, Università degli Studi di Firenze, 50144 Firenze, Italy; ada.baldi@unifi.it (A.B.); campeggistephanie@gmail.com (S.C.); anna.lenzi@unifi.it (A.L.); 2 Departamento Biología Aplicada, Escuela Politécnica Superior, Universidad Miguel Hernández, Ctra. Beniel Km 3.2, 03312 Orihuela, Spain; t.egeamolines@gmail.com (T.E.); cobon@umh.es (C.O.); 3 Departamento Biología Vegetal, Campus de Espinardo, Universidad de Murcia, 30100 Murcia, Spain; drivera@um.es

**Keywords:** ethnobotany, traditional gastronomy, vegetables, fruits, nutrients, food safety, medicinal plants, domestication

## Abstract

This paper provides an overview of wild food plants traditionally used in the gastronomy of Tuscany, an Italian region with high biological diversity and whose cultural heritage is well known. Forty-nine bibliographic sources, including five unpublished studies, were reviewed. A list of species with ecological characteristics, plant parts used, use category (food, liquor, or seasoning), methods of preparation (raw or cooked), and recipes is presented. The use of 357 taxa (3711 use reports, URs), was recorded, belonging to 215 genera and 72 botanical families. Over the total taxa, 12 are new for Tuscany, 52 seem not to be present in other Italian regions, and 54 were not detected in the consulted European ethnobotanical literature. Of these taxa, 324 (3117 URs) were used as food, while 49 (178 URs) and 81 (416 URs) were used for liquor and seasoning, respectively. Of the 17 different food recipes, cooked vegetables constituted the largest group, followed by salads, omelets, snacks, and fillings. The chemical composition of the recorded food plants and the possible safety risks associated to their consumption, as well as their traditional medicinal use, are also shown. This review highlights the richness of ethnobotanical knowledge in Tuscany. Such biocultural heritage can be a “source of inspiration” for agriculture. As a reservoir of potential new crops, wild edible flora may contribute to the development of emerging horticultural sectors such as vertical farming and microgreens production. Moreover, the nutrient content and healthy properties of many wild food plants reported in this study has the ability to meet consumer demand for functional foods.

## 1. Introduction

Consumption of wild food plants has often been ignored and marginalized by modern agricultural production systems, as it is considered an emergency practice to integrate an otherwise poor diet during times of food shortage due to war or crop failure [1,2,3,4]. This vision is even reflected in the terminology; adjectives such as “underutilized”, “neglected”, “orphan”, “minor”, “promising”, “niche”, “local”, and “traditional” are often used to describe these species [5]. Although “marginalized, if not entirely ignored, by researchers, breeders and policy makers” [6] these plants are recently gaining new attention; according to Schulp et al. [7], about 14% of the EU population collect—at least occasionally—wild food plants and mushrooms intended for home consumption or informal marketing. A revival of interest is especially rooted among the more highly educated young or middle-aged classes [8] and it is part of a process aimed at rediscovering the local cultural heritage associated with regional culinary traditions [9]. Several chefs have begun to use wild vegetables to experiment with different tastes and textures in their recipes, reinventing culinary traditions with the proposal of new local gastronomies [10]. Local fairs and specialized markets have been following the “wild-green-centred cuisine” wave for a few years, while thematic courses for on-field identification and training in the culinary uses of wild greens have recently been multiplying [9,11]. Discovering that many of these plants can play an important role in a healthy diet as an alternative source of minerals and vitamins, as well as antioxidant secondary compounds [12,13,14,15] and essential oils [16], was a turning point. Their use was first promoted by health-oriented people in the framework of a healthy lifestyle, but now it is suggested as a part of a new food strategy to manage malnutrition problems [5] and diversify the human diet [17], particularly in local food systems. According to the Global Nutrition Report 2021 (https://globalnutritionreport.org/reports/2021-global-nutrition-report/) (accessed on 17 November 2021) about 2 billion people lack key micronutrients, and 88% of countries face malnutrition. A substantial proportion of European children suffer from micronutrient malnutrition due to a dietary deficiency of vitamins (vitamin D and vitamin E in particular) and minerals [18]. Three out of four deaths in adult age are caused by diet-related diseases such as diabetes and hypertension, particularly in emerging economies and in low-to-middle income countries (https://globalnutritionreport.org/reports/2021-global-nutrition-report/) (accessed on 17 November 2021). COVID-19 is worsening the overall prospects, particularly in low- and middle-income classes, due to food shortages and the deteriorating quality of diets. Good nutrition is a key element in the defense against COVID-19 as undernourishment has been proven to weaken the immune system, exposing individuals to a greater risk of severe illness due to infection [19]. On the contrary, provitamin A, vitamin C, and other antioxidants which are contained in some wild food plants in high amounts are particularly useful for boosting the immune system [20]. Some authors suggest that wild plants could also be used in low-input sustainable food production systems in marginal areas [21,22], allowing an increase in the production potential of agroecosystems [17]. The majority of these plants grow in disturbed sites such as farmlands, inhabited areas, and borders of paths and roads. They are adapted to withstand stressful conditions typical of marginal lands where grazing is widespread and arable agriculture consists of low-input traditional cultivation methods [23,24].

According to Hadjichambis et al. [25], 2300 different wild plants and fungi taxa are still gathered and consumed as food in the Mediterranean Basin. These species are still considered to be a fundamental aspect of local culture in the rural areas of Italy; thanks to their taste and nutritional benefits, they are common ingredients in many typical and traditional dishes [26]. Every Italian region counts numerous local plant-based recipes which often differ substantially from village to village, and the same species can be cooked in various ways, some of which have remained almost unchanged since ancient times [27,28,29]. Many of these plants also have ethnomedicinal uses [30].

**Figure 1:**
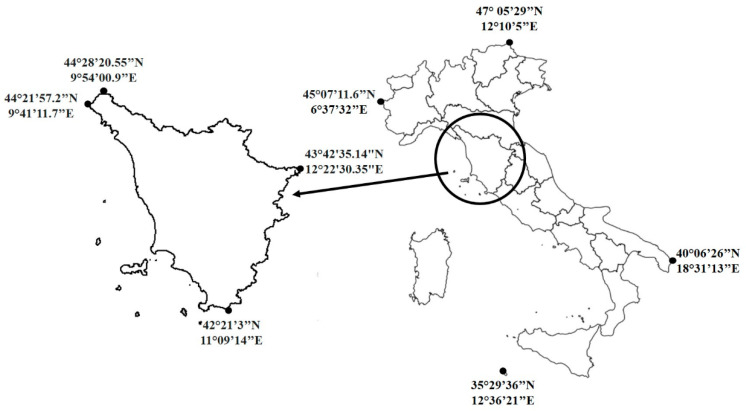
Study area: Tuscany region (Central Italy).

Tuscany is a central region of Italy with an extension of about 23,000 km^2^ (Figure 1). The strong climatic, topographic, and edaphic variability may explain the high diversity of vascular plants found in this region. Its rich flora counts about 3000 native taxa, around 3% of which are endemic [31]. Although several studies have been carried out on wild food plants gathered and consumed in the traditional regional dishes, no attempts have been made yet to carry out a global analysis on this subject. The aim of this research is to fill this gap in research providing a meta-analysis of the Tuscan wild vegetables used as foods, liquors, and seasonings. We also update this ethnobotanical information by including original data from studies carried out by our research group in areas previously understudied. With this work we intend: (i) to provide a complete catalogue of wild plants used in traditional Tuscan gastronomic culture, recording the taxa and the food uses associated with them, and identifying the most important ones; (ii) to build a database accessible to the international scientific community for the promotion of cross-cultural and geographical analysis of plant use patterns. The majority of the collected information is reported in local publications with low diffusion and popular-level books, most of which are written in Italian and not indexed in international databases (so-called “grey literature”). The difficulty of finding bibliographic sources and language constraints can be a “building block” of sorts for further cross-analyses [32]; (iii) to explore how many of these species can be considered as nutrient-rich and healthy foods. For this purpose, we have collected information on the nutritional composition and toxicity available in literature for the recorded plants. We have also highlighted which species are reported as medicinal plants in the ethnobotanical literature, specifying the medicinal use and whether the same part of the plant has both a medicinal and food use. The paper concludes with some perspectives on the introduction of wild plants into agricultural production systems.

## 2. Materials and Methods

### 2.1. Bibliographic Sources

A preliminary search of references concerning the use of wild food plants in Tuscany was carried out, consulting the sources cited in Tomei and Trimarchi [33] and Guarrera [34]. A further search was conducted through the main databases (Web of Sciences, Scopus, and Google Scholar) by using the following keywords: “etnobotanica Toscana”, “ethnobotany Tuscany Italy”, “piante alimentari spontanee Toscana”, “wild food plants Tuscany Italy”, “piante alimentari tradizionali Toscana”, “Traditional food plants Tuscany Italy”. Due to the abundance of material, the avoidance of repetition became crucial in order to obtain accountable results. We extracted data only from primary research (information collected by the authors in the investigated area) reported in different sources (scientific papers, popular books, research reports). We excluded data from systematic reviews/meta-analyses unless these data concerned taxa, areas, or uses not reported in the primary research. We also considered original data collected by our research group and included in graduation theses. Overall, the ethnobotanical sources which are considered reliable amount to 49, of which 20 are books (19 in Italian and 1 in Spanish), 20 scientific papers (16 in Italian and 4 in English), 4 conference proceedings (all in Italian), and 5 theses (all in Italian) (Supplementary Material S1). Ethnobotanical literature on the use of wild plants from Italy and other European countries has also been cited in this work as a comparison tool (Supplementary Material S1). 

### 2.2. Taxonomy

In the consulted literature, we considered only records reporting the full name of the species/subspecies, excluding incomplete and ambiguous information; for example, *Mentha* sp. or *Mentha* sp. pl. were explicitly excluded. The nomenclature and taxonomy of the different taxa was uniformed according to Bartolucci et al. [31] and Galasso et al. [35].

### 2.3. Native or Alien Plants

In the context of this review, plants were considered wild when, even if cultivated, they were listed as native (N) to Tuscany in Bartolucci et al. [31] or as alien (A) spread in the natural environment [35]. According to Galasso et al. [35], alien plants were further distinguished in Archaeophytes (Ar), Neophytes (Ne), Naturalized (Nat), Invasive (Inv), Feral (Fer). The alien plants reported by Galasso et al. [35] as Casual (i.e., plants seldom found outside cultivation and unable to self-maintain) have been excluded from this research.

### 2.4. Data Organization

All the collected data were filed in a database (analytical table) consisting of a spreadsheet (Windows Excel 2013). Each row is a distinct “record” (i.e., all the used plant parts and all the recipes reported for a single species by a single bibliographic source for a specific area). Each single plant part or each single recipe included in each record was considered as one use report (UR). We distinguished the following plant parts categories: (1) epigeal organs, for the whole aerial section of the plant or, more specifically as (2) bark, (3) branches, (4) flowers, including buds and petals, (5) fruits, for fruits, infructescence, and false fruits, (6) leaves, (7) seeds, (8) shoots, and (9) stems. The underground parts such as roots, bulbs, and tubers were collectively classified as (10) hypogeal organs. Lastly, the use of all parts was indicated as (11) whole plant. Data concerning the use of the reported species was organized according to a hierarchical approach. We established three main categories of general use: food, liquor, and seasoning. In the seasoning category we considered all the aromatic plants regardless of whether they were used in food preparation or added after food is served. Secondly, two forms of consumption, raw food and cooked food, specified in which way food plants were consumed. The general food use was classified into the following 17 different categories: (1) beverages (recreational tea, juice, syrup), (2) coffee substitute, (3) cooked fruits, (4) cooked vegetables (species cooked as single or mixed with others, and soups), (5) flour substitute, (6) fried, (7) jam, (8) omelets, (9) pasta/dumplings, (10) raw fruits, (11) pickles, (12) quiche filling, (13) ravioli filling, (14) risotto, (15) rural snacks (plants consumed directly in the fields, outside of regular organized meals), (16) salads, and (17) sweets.

### 2.5. Nutritional Properties, Toxicity, and Medicinal Uses on

We carried out a bibliographic study on the chemical composition (macro- and micronutrients) and the possible toxicity/presence of antinutritional factors in the food plants reported in our study. We also investigated their traditional medicinal uses, distinguishing between internal (i.e., when oral intake of the drug is required, such as infusion or decoction) and external (i.e., when the drug is externally applied, such as an ointment on the skin). The ailments were categorized with the International Statistical Classification of Diseases and Related Health Problems (ICD) from the WHO (https://icd.who.int/browse11/l-m/en) (accessed on 14 October 2021).

### 2.6. Data Analysis

The Portéres Ethnobotanicity Index (EI) [36], which consists of the percentage ratio between useful plants and total flora growing in a geographic area, has been used to assess the importance of wild food plants in the region. We considered both total Tuscan native and alien flora (excluding alien casuals) as reported in Bartolucci et al. [31] and Galasso et al. [35], respectively. The Cultural Importance Index (CIs, the sum of the proportion of bibliographic sources that mention the use of each species) was calculated for each species to identify the most valued plants in the local gastronomy [37]. This index takes into account both the total number of URs for each species and its versatility, i.e., the diversity of its uses. A Cultural Importance Index for each use category and each food recipe (CIu, the sum of the proportion of bibliographic sources that mention a particular use of all the species) was also calculated to assess the cultural importance of the reported uses [38]. We estimated the Chao1 index as a measure of richness and Shannon (H) and Simpson indices as diversity metrics of each use general category and each recipe. Chao-1 is a measure of total richness and is particularly useful for data sets that include low-abundance species, as is the case with some recipes in this review. The Simpson index gives more weight to common or dominant species, meaning that a few rare species will not affect the diversity of the sample. Finally, the Shannon index reflects species numbers and evenness of species abundance. Approximate confidence intervals for all these indices were computed with a bootstrap procedure. Cluster analysis was used to identify homogeneous groups of different uses based on a matrix of presence/absence of the recorded species within each recipe, including liquor and seasoning uses. We carried out the agglomerative dendrogram using the Euclidean distance and average method, since it had the highest cophenetic correlation coefficient (0.9504). Bootstrap analysis of clustering was performed using 1000 replications. The software PAST 4.08 was employed to calculate diversity indices and to perform cluster analysis.

## 3. Results

### 3.1. General Data

Overall, 2378 records and 3711 Urs for a total of 357 taxa, belonging to 215 genera and 72 botanical families, were counted (Supplementary Material S2). The thirty most important taxa are shown in Table 1. These data clearly highlight the importance of wild food plants in the cultural tradition of the Tuscan region, compared to other areas of different extension. For example, Guarrera [34] reported a total of 580 food species for the whole of Italy, while Guarrera and Savo [29] stated that 276 wild taxa are used in traditional vegetable mixtures in Italy. More recently, the review carried out by Paura et al. [39] reported the use of 1103 wild food taxa for the whole of Italy. Biscotti et al. [40] and Pasta et al. [41] summarized the ethnobotanical use of 214 and 292 wild food taxa for the Apulia and Sicily regions, respectively. Tardìo et al. [42] in their review reported the use of 419 edible species for the whole of Spain. In the northwestern region of the Iberian Peninsula, Pardo-de-Santayana et al. [43] mentioned the use of 97 wild food species while Gras et al. [44] reported 278 taxa (beverage excluded) for the Catalan linguistic area. In their comparative analysis on the wild food plants gathered in the Mediterranean Basin, Leonti et al. [45] identified 84 species for Italy (Castelmezzano in Basilicata and Gallicianò in Reggio Calabria), 147 for Greece (mostly Crete) and 173 for Spain (Cuenca, Albacete, and Murcia provinces). Another Mediterranean country where the use of wild plants as food is widespread is Turkey (e.g., 154 taxa in the Iğdır Province [46] and 61 taxa in the Bingöl area [47]).

In our study, the EI was 10.3% considering only native and alien wild food plants; it was slightly lower (10.2%) when only native flora was taken into account. These values are consistent with those of other Italian regions (range 5.4–11%) for seasonings only [48], or different areas of the Iberian Peninsula (range 5.05–29.01%) for all food and medicinal uses [49]. Recently, Gras et al. [44] have reported that 6.62% of the total native flora in the Catalan linguistic area has a traditional food use.

Moreover, our results provide a relevant and novel contribution to the knowledge of food plants in Tuscany and in Italy by including original data from graduation theses. It is important to point out that the review carried out by Paura et al. [39] counted a total of 341 food species for Tuscany. Unfortunately, this database is not accessible online and it was not possible to compare our data to those recorded by these authors. Twelve species included in our study (Supplementary Material S2) were not previously reported in the consulted literature concerning Tuscan ethnobotany, and three of them are new for Italy [*Clinopodium alpinum* (L.) *Merino*, *Digitalis lutea* (L.) and Oxalis debilis Kunth]. Among all the records included in our database, 52 regarded taxa not reported for Italy and 54 regarded taxa not reported for Europe (Supplementary Material S2). Among the species reported in our study, 158 were also recorded in Sicily, 148 in Campania, 140 in Basilicata, and 135 in Sardinia. Less correspondence was found with Northern Italy: Friuli Venezia Giulia (118 common species), Lombardy (92), Piedmont (85), Emilia-Romagna (59), and Veneto (34). By comparing our dataset with the European literature, we found the highest correspondence with Spain (203 common species), followed by Bosnia-Herzegovina (188) and Croatia (158). The observed similarities, both at the Italian and European level, are difficult to explain; we can hypothesize that ecological and floristic causes, as well as sociocultural factors, may have contributed to this pattern of shared taxa. Furthermore, the different number of ethnobotanical studies carried out in different areas and their varying degrees of detail may have affected the described results.

As commonly observed in the ethnobotanical literature (see for example [44]), similarly in our study, most species (50%) were reported by fewer than three sources. These plants are below the threshold fitting the reliability requisites indicated by Johns et al. [50]; however, we believe that these species should not be overlooked as food alternatives. A low number of reports could depend on the different number of studies conducted in different geographical areas with different ecological characteristics rather than to a scarce edible value of the species. In this regard, the use of *Crithmum maritimum* L., a perennial halophyte common in the coastal areas of the Mediterranean region, is exemplary; although the species is known as an emerging crop in many European countries [51], it was reported as food plant in Tuscany with only three citations. This is due to the low number of ethnobotanical studies carried out in the Tuscany coastal area.

### 3.2. Botanical Families

The majority of the families (69%) were reported with only one (27 families), two (14 families), or three species (9 families). With 60 taxa (521 records), Asteraceae represented 16.5% of the investigated ethnoflora. Other highly represented families were Lamiaceae (33 taxa; 275 records), Rosaceae (33; 219), Brassicaceae (21; 97), Apiaceae (14; 133), Amaryllidaceae (14; 46), and Fabaceae (14; 47) which collectively represented 36% of the listed taxa. These families are among the most important of the Tuscan flora, with a high number of taxa [31], and appear as the most relevant families in several ethnobotanical studies in Italy [8,41] and in the Mediterranean area [42,45,52]. A regression analysis was carried out between the number of edible wild species recorded in this paper and the total number of both native and alien species per family in the Tuscan Flora. The results showed that families with high positive residuals (i.e., those families having a number of reported food species significantly higher than would be expected under random selection conditions) were Lamiaceae (20.8), Rosaceae (20.1), Asteraceae (18.6), Brassicaceae (7.2), Amaryllidaceae (5.4), and Apiaceae (1.4). It is also worth noting that these families include many plant species cultivated for food purposes [45]. Underrepresented families in our study (i.e., those families having significantly negative residuals) were Poaceae (−27.6), Fabaceae (−11.2), and Orchidaceae (−9.2). Similar results have been reported for the edible wild vegetables in Spain [52]. Specifically, Poaceae showed a very low number of wild food species (2), even if this family is the second richest of the Tuscan flora [31]. The low number of Poaceae taxa is a common finding in ethnobotany of the Mediterranean area [41,45]. Several studies have shown that wild Poaceae could have played an important role in hunter-gatherer subsistence strategies [53,54]. A progressive rapid decrease in the use of these small-seeded grasses has been associated to the difficulty of gathering them and to their fibrous husks, making these grains difficult to process and eat compared to domesticated naked grains. Nevertheless, the food use of wild Poaceae survived in Africa, Asia, and Australia [53].

### 3.3. Life Forms and Ecological Characteristics

According to the Raunkiaer life forms, hemicryptophytes were dominant (40%), followed by therophytes (20%) and phanerophytes (20%). Geophytes were 12%, while chamephytes and lianas were 6% and 2%, respectively. The predominance of hemicryptophytes (both perennial and biennial herbs) on therophytes (annual herbs) is a common finding in ethnobotanical studies; on the other hand, people favored the selection of those species which were easy to collect year after year in a given place [52]. Most hemicryptophytes and therophytes were scapose and secondarily rosulate; they are plants widely spread in dry and strongly human-affected habitats. In particular, rosulate plants are common in areas where grazing by livestock causes strong disturbance due to trampling and mechanical injury to leaves. The relatively low number of edible geophytes could be explained by the high presence of poisonous species in this biological form [41]. Since wild plants are usually well adapted to the climatic and edaphic conditions of where they evolved, their use and their possible cultivation could imply a sustainable diet system [55] less dependent on external inputs and more attentive to the use of water resources and to the conservation of physicochemical soil properties. The wild food plants recorded in this study that showed high tolerance to heat, drought, nutrient-poor conditions, and soil salinity are reported in Table 2. Specifically, one species is well adapted to heat (*Rumex bucephalophorus* L.), and four species (*Atriplex halimus* L., *Crithmum maritimum*, *Echinophora spinosa* L., and *Teucrium montanum* L.) are well fitted to dry conditions. Three species (*A. halimus*, *C. maritimum*, and *Salicornia perennans* Willd. subsp. *perennans*) are salt-resistant and are able to complete their development in high-saline environments.

### 3.4. Native or Alien Plants

Most taxa consumed in the Tuscan region are native (327 taxa; 92%). This finding suggests that people commonly collect plants that are more embedded in the local culture, and they give priority to ethnobotanical uses which they are more familiar with. Edible native plants play an important role in supporting the process of food sovereignty, allowing people to take control of the food supply and consumption [56,57]. In this regard, conservation and valorization of this biocultural heritage is a crucial step on the path towards alternative visions and practices that can help to build more socially equal and sustainable food systems [55]. Out of the 30 aliens, 20 were archaeophytes and 10 neophytes. As pointed out by Nuñez et al. [58], the time of introduction can strongly affect the perception and the knowledge of people about the use of alien species; in this regard, archaeophytes had more time to be experimented with and possibly be included in the local sociocultural context. Moreover, 12 of these archaeophytes are feral; cultivated for a long time [e.g., *Malus domestica* (Borkh.) Borkh., *Punica granatum* L. or *Prunus* species], they coevolved biologically and culturally with human communities. It is interesting to notice that six neophytes are invasive and that two of them [*Crepis sancta* (L.) Bornm. subsp. *nemausensis* (P.Fourn.) Babc. and *Robinia pseudoacacia* L.] are reported in several sources (Supplementary Material S2). The introduction of *C. sancta* was reported for the first time in Tuscany in 1827 but it rapidly spread in ruderal and human-affected areas. *R. pseudoacacia* was introduced in Tuscany in 1788, spreading and becoming a common feature of the lowland and hilly woods. The quick incorporation of these invasive species into local gastronomy probably lies just as much on their rapid and wide diffusion as on their abundance and easy visibility [59]. Therefore, they are perceived as intrinsic components of the local landscape. For example, Robinia woods are not considered an alien part of the landscape by public opinion across Europe [60] and the tree is used for culinary uses and other important services. On the other hand, local traditional gastronomic culture often proved to be permeable and able to evolve, strongly susceptible to the influence from other cultures, and ready to integrate new plants or new recipes, allowing to diversify and improve the diet [57].

### 3.5. Use Categories and Taxa

**Figure 2:**
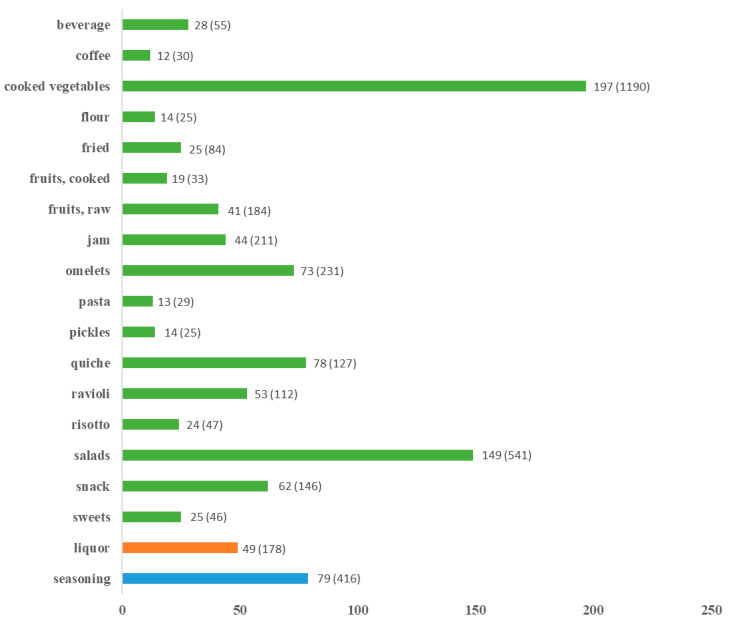
Number of species and (Urs) for each detailed use.

Taxa used as food were 324 (3117 URs; CIu: 63.61); taxa used for production of liquor were 49 (178; 3.61); while taxa used as seasoning were 81 (416; 8.48) (Figure 2). 

Plants mentioned exclusively as food were 248, while those mentioned exclusively as liquor were 5. Plants used only as seasonings were 22. Vegetables were represented by 284 taxa (2952 URs), of which 220 plants were used as leafy greens (“plant species of which the leafy parts, which may include young and succulent stems, flowers and very young fruits, are used as a vegetable” [61]) and 14 exclusively as root vegetables (including species with modified stems). Fruit species were 65. The most frequently reported (top URs) and the most culturally important (top CI values) taxa were vegetables (Table 1). The CI index of vegetable plants was 51% of the total CI, followed by seasoning plants (18%), liquor (17%), and fruits (14%). The most important species was *Borago officinalis* L. (106 Urs; Cis: 1.58), followed by *Urtica dioica* L. subsp. *Dioica* (97; 1.45), *Taraxacum* FH. Wigg. Sect. *Taraxacum* (81; 1.21), *Cichorium intybus* L. (79; 1.18), *Silene vulgaris* (Moench) Garcke (77; 1.15), *Sonchus oleraceus* L. (70; 1.04), *Foeniculum vulgare* Mill. (68; 1.01), and *Papaver rhoeas* L. subsp. *rhoeas* (65; 0.97). These data are in accordance with Ghirardini et al. [8] and Hadjichambis et al. [25] for Italy and with several authors for the Mediterranean area [25,42,45,62], proving the existence of a well-established pattern of use of these plants.

To validate the information recorded in our study, we checked how many and which plants of the Tuscan edible ethnoflora are included in the Plants for a Future database (PFAF) (https://pfaf.org) (accessed on 8 September 2021). Out of the 357 taxa cited in this research, 286 are reported in the PFAF database as edible. Of these, 22 have the highest edibility rate (5) and 43 have a rather high edibility rate (4). A total of 64 species have the lowest edibility rate (1). We found a significant positive Spearman correlation (*R* = 0.2536; *p* < 0.050) between the edible value as indicated in the PFAF database and the number of records for each species in our study, suggesting that the edible value is an important factor affecting the use of a species. The popularity of a plant is a function of complex dynamics of intertwined factors [8,29,44,45]. Compared to fruits, vegetables often require more time-consuming preparation (grading and separation of the useful parts from the rest of the plant, cleaning, washing, and frequently cooking) and sometimes the use of additional ingredients (oil, salt, garlic, and others). However, wild vegetables offer a wide combination of tastes, flavors, and textures that can strongly affect the individual’s consumption preferences [8], expanding the gastronomic offer. For example, Asteraceae have a bitter taste, varying in intensity not only among the different species [63] but also among the different plant parts [64]; meanwhile, aerial parts of Brassicaceae are reported to have different grade of spiciness [8]. The organoleptic characteristics are associated to different chemical substances such as saponins (acrid and bitter taste), organic acids (acid taste), alkaloids and tannins (bitter and astringent taste), sugars, d-amino acids, and small-l amino acids (sweet taste). Terpenoids and their derivates are aromatic substances determining the smell of many plants [16]. Terpenoids and their derivates are aromatic substances determining the smell of many plants. Chemosensory perception is an important tool to detect the edible and medicinal characteristics of plants [65,66] and depends on both biological and cultural processes [65]. Bitterness of some vegetables is generally appreciated, as it is often associated with a folk perception of healthiness [67,68]. Most of the reported vegetables are species growing in proximity to human communities; people prefer to collect plants easily available and growing close to them [69,70]. Economic factors [44] can also be considered for explaining the popularity of some vegetables or fruits consumed raw or processed (e.g., jams and syrups); these products, sold on local markets, become a source of supplementary income for families. 

### 3.6. Plant Parts

**Figure 3:**
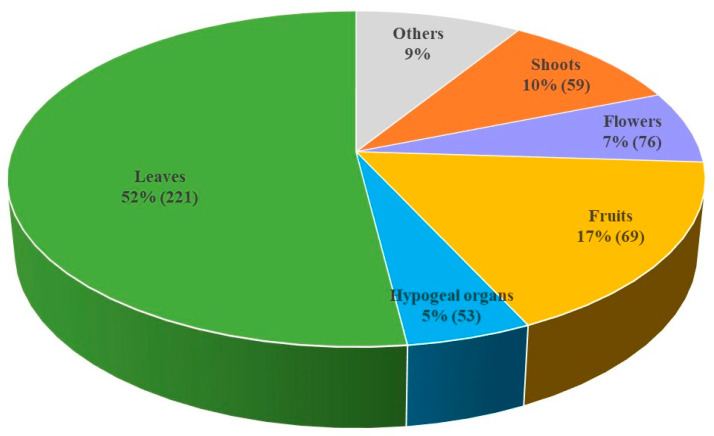
URs% and (number of species) for the most cited plant parts.

The most used parts were leaves (2098 Urs), which were reported three times more than fruits (695) and five times more than shoots (395) (Figure 3). Flowers and hypogeal organs had 274 and 207 Urs, respectively (Figure 3). The remaining parts resulted to be used less frequently, ranging from 121 Urs for seeds to one UR for bark. Leaves are often the main ingredient for salads, soups, and vegetable mixtures. Moreover, they are frequently used to flavor dishes. Flowers are used to decorate salads or pasta dishes (e.g., *Bellis perennis* L., *Borago officinalis*, and *Robinia pseudacacia*) or as a snack (*Primula vulgaris* Huds. subsp. *vulgaris*, *Oxalis curniculata* L., *Lamium maculatum* L.). The fruits are employed for making jams [e.g., *Hippophae fluviatilis* (Soest) Rivas Mart., *Rosa canina* L., *Sambucus nigra* L.] and liquors (e.g., *Arbutus unedo* L., *Cornus mas* L., *Juglans regia* L.). Fruits are also consumed raw (among others, *Prunus* sp.pl., *R. canina* and *Vaccinum myrtillus* L.), cooked (e.g., *C. mas*, *S. nigra*, *Ziziphus jujuba* Mill.), as a flour substitute (*Castanea sativa* Mill., *Rosa* sp.pl.), or a coffee substitute (e.g., *Quercus* sp.pl.). Hypogeal organs (roots, bulbs, rhizomes, and tubers) are used in several different preparations. Roots of *Cichorium intybus* and *Taraxacum* sect. *Taraxacum* are traditionally used as a coffee substitute; bulbs of *Muscari comosum* (L.) Mill. and *Allium* sp.pl. are eaten cooked or raw, while roots of *Campanula rapunculus* L. and *Tragopogon porrifolius* L. are an ingredient of salads. Shoots are used in salads and omelets (e.g., *Urtica dioica* subsp. *dioica* and *Clematis vitalba* L.). A factor explaining the clear preference of aerial parts is that they are easier to collect compared to hypogeal parts and people are more familiar with them, being the most visible parts [44]. 

The preference of some parts over others may depend on the different concentration of chemical compounds strongly affecting the taste or scent. For example, *C. rapunculus* roots are sweet while the leaves are rather bitter, and *Tragopogon pratensis* L. leaves are bitter while the root has a walnut taste [64]. The opposite occurs for *Crepis leontodontoides* All. [63]. Moreover, vegetative organs are harvestable in different seasons, while flowers, fruits, or seeds can be collected only in the reproductive season, limiting their consumption to a short period. Leaves are especially collected at the beginning of spring when they are soft and rich in bioactive compounds (vitamins, anthocians, flavones) and can be eaten raw. When cooked, soft leaves are preferred to coarse leaves, which require longer cooking times.

### 3.7. Preparation Forms and Detailed Uses

Wild plants were found to be consumed primarily in their *cooked* form; in fact, out of the 326 taxa having a food use, 102 are solely consumed *cooked*, while 54 plants are exclusively consumed in their *raw* form. A total of 170 are consumed both cooked and raw. These results agree with other studies [25,71] reporting that wild food plants are commonly cooked in Italy. Cooking is often used to make edible or more pleasant the gathered parts (i.e., to soften fibrous epigeal or hypogeal organs) and/or to reduce the content of undesirable compounds or potentially toxic substances. Variation in the modalities of preparation can also be due to different traditional patterns of use occurring at national and regional scales. In Croatia and in Spain, most wild greens are boiled or fried [71], while in southern France [71], as well as in the Spanish–Catalan linguistic area [44] they are often eaten raw. Pieroni et al. [28] pointed out that the raw consumption of wild edibles in a south Italian village was rare as a result of recent cultural changes; on the contrary, other studies conducted in Italy [64,72] reported that wild plants were mainly used as salads.

As shown in Figure 2, cooked vegetables were the most cited category of consumption (197 species; 1190 URs; CI_u_: 24.28) followed by salads (149; 541; 11.06), omelets (73; 231: 4.71), snacks (62; 146; 2.97), quiche filling (78; 127; 2.59), and ravioli filling (53; 112; 2.28). Fruits consumed raw were reported for 41 species (184 URs; CI_u_: 3.75) while 19 species (33; 0.67) were counted for cooked fruits. Finally, fruits from 44 species (211 URs; 4.30) were used to make jam. Other less frequently reported food uses were fried (25 species; 84 URs; CI_u_: 1.71), beverage (28; 55; 1.12), risotto (24; 47; 0.96) sweets (25; 46; 0.94), dumpling or pasta (13; 29; 0.59), and pickles (14; 25; 0.51). Flour and coffee substitutes are uses associated to the war or famine periods, and they remain only as a memory for the more elderly; they counted 14 (25 URs; CI_u_: 0.51) and 12 (30; 0.61) species, respectively (Figure 2).

A total of 122 taxa had only one detailed use; in particular, 36 plants were used only as cooked vegetables, 19 only as snacks and 14 only as salads. The most versatile species were *Castanea sativa* and *Rubus ulmifolius* Schott. (10 different uses); *Borago officinalis*, *Clematis vitalba*, *Rubus idaeus* L. subsp. *idaeus*, *Sambucus nigra* (9 uses); *Rosa canina*, *Taraxacum* sect. *Taraxacum*, and *Urtica dioica* subsp. *dioica* (8 uses) (Supplementary Material S2). Compared to other Italian regions, vegetable soups are a feature of Tuscan traditional gastronomy [73,74]: 28 different recipes comprising a total of 92 wild vegetable taxa have been reported in the consulted literature. Some soups have only one wild vegetable ingredient (e.g., *B. officinalis* in the “burbugnon” soup); others have up to 30 different wild species (e.g., the “minestrella of Gallicano”) and have a complex composition comparable to other traditional soups such as “prebuggiun” in Liguria [75] or “pistic” in Friuli [76]. Several wild greens, in particular their tender basal rosettes of leaves or their whorls, are consumed raw in salads. The habit of consuming wild plants as rural snacks has been observed for other Italian regions [72,77,78] and for other Mediterranean countries [38,79]. This use included consumption of raw fruits (e.g., *Prunus spinosa* L. subsp. *spinosa* and *R. canina*), raw vegetables (e.g., the sour-tasting leaves of *Oxalis acetosella* L. and *Rumex* sp.pl.; the shoots of *R. canina* and *R. ulmifolius*; the hypogeal organs of *Gentiana acaulis* L. and *Polypodium vulgare* L.), the twigs of *Buxus sempervirens* L. and *Ostrya carpinifolia* Scop., and flowers sucked by children for their sweet nectar (e.g., *Lonicera caprifolium* L.; *Lamium* sp.pl. and *Oxalis* sp.pl.) (Supplementary Material S2). Omelets are another important way of cooking wild plants; leaves (e.g., *Bellis perennis* and *Silene vulgaris*), flowers (e.g., *Cercis siliquastrum* L. subsp. *siliquastrum* and *Primula* sp.pl.), shoots [e.g., *Helosciadium nodiflorum* (L.) W.D.J.Koch subsp. *nodiflorum* and *Ruscus aculeatus* L.], and hypogeal organs [e.g., *Bellevalia romana* (L.) Sweet and *Muscari* sp.pl.] can be added raw or blanched in boiling water to the omelet mixture (Supplementary Material S2). Leaves of some vegetables are added to the pasta dough (e.g., *Plantago* sp.pl. and *U. dioica* subsp. *dioica*) or used to fill ravioli [e.g., *Blitum bonus-henricus* (L.) Rchb. and *Malva* sp.pl.] (Supplementary Material S2). The same species are also often added to the dough used to fill quiches or used to dress risotto. The most popular flour substitute, especially in mountain areas, was raw *C. sativa* powder, which is still widely used today as an ingredient of soups, stews, and polentas, and to make cakes and bread. Other flour substitutes were obtained from hypogeal organs (e.g., *Arum italicum* Mill. subsp. *italicum* and *Arundo donax* L.) and seeds [e.g., *Fagus sylvatica* L. subsp. *sylvatica* and *Lolium pretense* (Huds.) Darbysh.]. Among beverages, recreational teas (*sensu* [80]) are reported with 17 species. They are infusions made with roots, leaves, flowers, or fruits and mainly drunk for their taste and smell; however, they are also locally known for their healthy attributes. Other species (e.g., fruits of *R. idaeus* subsp. *Idaeus* and *Vaccinium myrtillus* L.) are used to make juices or syrups. *Quercus* acorns were commonly used as a coffee substitute; other species reported for this category are *Cichorium intybus* (roots), *Arctium lappa* L. (roots) and *Vitis vinifera* L. (seeds). The most popular species whose fruits are consumed raw (sometimes cooked) or in the form of jams are those from *Arbutus unedo* (32 URs), *R. canina*, *Fragaria vesca* L. subsp. *vesca*, and *S. nigra* (23) (Supplementary Material S2). The number of taxa used as seasoning in our study (79) was much higher than that listed by Motti [48] for Tuscany itself (24) and for other regions such as Campania (23), Abruzzo, and Lombardy (22 each). Most of these plants belong to Lamiaceae, Amaryllidaceae, and Apiaceae. The high number of species used to make liquors (49) confirms that traditional alcoholic beverages are an important part of the Tuscan food culture [81,82]. There are different types of alcoholic beverages traditionally produced in Tuscany. For example, fruits of *P. spinosa* and *Sambucus ebulus* L. are distilled to produce “grappa”; those of *Cornus mas* and *Prunus* sp.pl., and the flowers of *Robinia pseudoacacia* are fermented (sometimes with addition of sugar and/or water) to prepare low-alcohol beverages; leaves of *Artemisia absinthium* L., roots of *Gentiana asclepiadea* L., flowers of *Viola odorata* L., and fruits of *Myrtus communis* L. are macerated in grappa or in 95% ethanol and mixed with a syrup made with sugar and water [82]. Finally, some plants (e.g., leaves of *A. absinthium*) are used to aromatize wine.

We observed a wide range of variation in the alpha diversity of ingredients used in different food categories. Results from the calculation of diversity parameters showed that cooked vegetables had the highest diversity values (H = 4.63; Simpson: 0.98) followed by salads (4.55; 0.98), quiches (4.42; 0.98), snacks (3.99; 0.97) and omelets (3.73; 0.95) (Table 3). 

**Figure 4:**
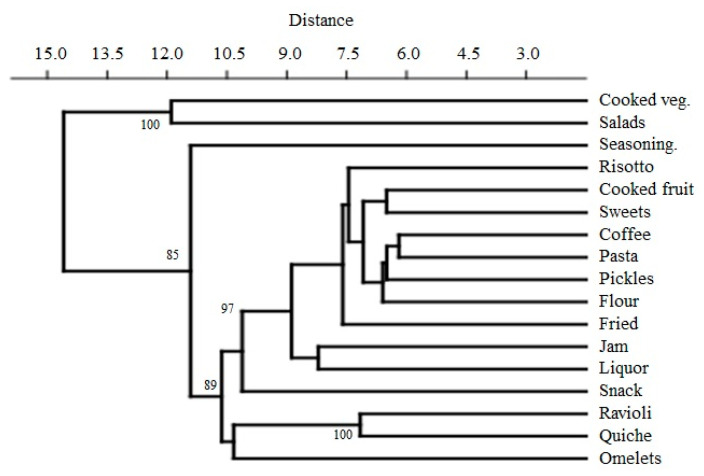
Cluster analysis of the different uses based on the presence/absence of taxa.

Chao1 index showed that the expected richness for each use is always above its observed value, suggesting that the recorded information about the use of plants in the different recipes was not enough to represent all the diversity present in the local ethnobotanical culture. All these data show that traditional dishes—in particular those entailing vegetables—are very diversified and offer a variety of gastronomic possibilities exploring different tastes, flavors, and textures. The hierarchical cluster showed a consistent distribution pattern, with the results revealing a taxa differentiation among six groups. Cluster I was composed by omelets; cluster II was formed by fillings (ravioli and quiches); cluster III by snacks; cluster V by seasoning; cluster VI by salads and cooked vegetables; cluster IV by all other uses (Figure 4).

### 3.8. Nutritional Value of the Reported Plants

From the bibliographic research, we obtained information on the nutritional values of 108 taxa. For 40% of them, it was possible to obtain a rather complete nutritional profile including proteins, carbohydrates, lipids, macro- and microelements, and Vitamin C. Table 4 shows the top five highest-ranked species. Most wild fruits and vegetables are rich in carbohydrates and relatively low in proteins and lipids, with the exceptions of the fruits/seeds from some tree species (*Pinus pinea* L., *Fagus sylvatica* subsp. *sylvatica*, *Juglans nigra*, *Corylus avellana* L., and *Castanea sativa*) and the leaves of *Atriplex hortensis* L. In terms of mineral composition, calcium occurs at the highest concentration in leaves of *A. hortensis* (2000 mg/100 g), potassium in leaves of *Bunias erucago* L. (2200 mg/100 g) and *Bellis perennis* (2053 mg/100 g), phosphorus in *P. pinea* seeds (508 mg/100 g), and magnesium in *Punica granatum* seeds (1697 mg/100 g). *B. perennis* (40.8 mg/100 g), *P. granatum* (32.3 mg/100 g), and *B. erucago* (24.1 mg/100 g) have an iron content much higher than spinach (2.7 mg/100 g) and meat (3.5 mg/100 g). Vitamin C content is high in the fruit pulp of *Hippophaë fluviatilis* (450 mg/100 g) and *Rosa canina* (426 mg/ 100 g); among vegetables, important sources of vitamin C are *Primula veris* L. (418 mg/100 g), *Primula vulgaris* (305 mg/100 g), and *Urtica dioica* subsp. *dioica* (285 mg/100 g), *Alliaria petiolata* (M.Bieb.) Cavara and Grande (261 mg/100 g; [83]), *Blitum bonus-henricus* (184 mg/100 g; [84]) (and *Sisymbrium officinale* (L.) Scop. (176 mg/100 g; [84]). Data on the availability of other vitamins is more sporadic and there are many species whose vitamin content is unknown. Provitamin A seems to be high in leaves of some vegetables such as *B. erucago* (962 µgRAE/100 g) [64], *Nasturtium officinale* R.Br. (665 µgRAE/100 g) [85], *Chenopodium album* L. subsp. *album* (580 µgRAE/100 g) [86], *Poterium sanguisorba* L. (556 µgRAE/100 g) [64], *Taraxacum* sect. *Taraxacum* (508 µgRAE/100 g) [86], and *U. dioica* subsp. *dioica* (476 µgRAE/100 g) [13]. High levels of vitamin B9 (total folates) can be found in *Asparagus acutifolius* L. (589 µg/100 g) [87], *Silene vulgaris* (519 µg/100 g) [87], and *Rumex pulcher* L. (506 µg/100 g) [14]. Vitamin E (α-tocopherol) is high in *Glechoma hederacea* L. (73 mg/ 100 g) [14], *Sinapis arvensis* L. subsp. *arvensis* (28 mg/100 g) [88], *Mentha pulegium* L. subsp. *pulegium* (28 mg/100 g) [14], and *Malva sylvestris* L. (20 mg/100 g) [13]. Out of the bioactive non-nutrients, natural phenolics have an important role in health promoting as nutraceuticals and as powerful anti-hepatotoxic agents [89]. Values, expressed as gallic acid, are highest for *M. sylvestris* (1692 mg/100 g) [13], *Crataegus monogyna* Jacq. (746 mg/100g), *Prunus spinosa* subsp. *Spinosa* (590 mg/100g), *Arbutus unedo* (542 mg/100 g), and *Rubus ulmifolius* (478 mg/100 g) [84].

All these data suggest that some wild food plants used in the traditional gastronomy of the Tuscan region could be targeted for systematic inclusion in the diet. However, some considerations must be taken into account. Firstly, the reported nutritional values are merely indicative and can vary considerably depending on the genotype, the environmental factors, the season of collection, and the developmental stage. For example, Oprica et al. [97] showed that vitamin C in *R. canina* fruits ranged between 274 mg/100 g and 449 mg/100 g along an altitudinal gradient. Lenzi et al. [98] found that minerals in *S. arvensis, P. sanguisorba,* and *Taraxacum* sect. *Taraxacum* varied significantly between different developmental stages. Ceccanti et al. [99] pointed out that leaves of *Cichorium intybus*, *Picris hieracioides* L., *Plantago coronopus* L., and *Rumex acetosa* L. cultivated in organic field conditions had higher levels of potassium and calcium than leaves of the same genotypes gathered in the wild. The nutritional profile may also depend on whether plant material has been processed and to what extent. Different cooking conditions are known to have an effect on some chemicals; for example, vitamin C and vitamin B9 are destroyed by heat, while most of the minerals are dissolved in the liquid cooking medium [87]. The preparation of the plants listed in our review seems to consider this; the plants having the highest vitamin C content are mostly used in the form of raw fruits (e.g., *Hippophaë fluviatilis*) or salads (e.g., *Primula veris* and *Primula vulgaris*).

Moreover, it is important to point out that bioavailability of some substances is often limited; the interaction between different nutrients may further affect the absorption, and therefore the quality, of some potentially highly nutritious wild food plants. Only less than 5% of iron from vegetable sources is absorbed in the gut [100] and some elements such as calcium and zinc are known to have an inhibitory effect on iron bioavailability [101]. Plants selected as iron sources must have low calcium content (for this reason, *B. perennis* should be excluded; Table 4). On the contrary, vitamin C has a strong effect in promoting iron absorption; it is desirable to associate plants characterized by a good level of iron availability with plants with high vitamin C (e.g., *B. erucago* and *Primula* sp.pl., leaves; Table 4). Calcium and magnesium are antagonists and should be in a ratio Ca/Mg from 2 to 4. *B. erucago* and *A. hortensis* have a balanced Ca/Mg ratio of 2:1 and 4:1, respectively, while *Plantago lanceolata* L. has an unbalanced Ca/Mg ratio (5.8:1) and could result in a magnesium deficiency.

### 3.9. Food Safety

A total of 169 plants listed in this paper were reported to have some kind of toxicity for humans according to the four categories reported in the TPPT database [102]: “very strong toxicity”, “strong toxicity”, “toxic”, and “weak toxicity”. For 83% of them we find a correspondence between the part of the plant that is toxic and the one that is consumed. People are generally aware of the toxicity of plants they gather and adopt strategies to limit the risk of possible adverse effects. The development stage is an important factor in affecting the toxicity of a specific plant part; for this reason, young shoots of *Clematis vitalba*, *Bryonia dioica* Jacq., and *Ruscus aculeatus* are preferred to the more mature ones, because they contain lower levels of toxic components. Another effective way to reduce toxicity is heat; 75 toxic plants out of 168 were solely consumed cooked, while 20 were recorded to be eaten raw, and 19 both cooked and raw. A total of 70% of species are toxic by ingestion, and 8% by contact; 22% are dangerous both by ingestion and contact. Some molecules such as sesquiterpene lactones (e.g., *Achillea millefolium* L., *Artemisia absinthium* and *Matricaria chamomilla* L.) and fucomarine (*Ammi majus* L. and *Ruta graveolens* L.) can cause dermatitis and light sensitivity. Plants having a “very strong toxicity” by ingestion were *Arum italicum* subsp. *Italicum, B. dioica, Buxus sempervirens, Digitalis lutea,* and *Taxus baccata* L. [102]. The alkaloid digoxin in *Digitalis* sp.pl. can cause nausea, vomit, abdominal pain, cardiac disturbances, and even death. Flowers of *D. lutea* were reported to be sucked as a snack in a mountain area near Florence (Supplementary Material S2). The same use was recorded for *Digitalis pupurea* L. in Sardinia [78] and *Digitalis thapsi* L. in Spain [42]. Steroidal alkaloids content of *B. sempervirens* is particularly high in leaves and bark; they can produce digestive and respiratory disorders, and even paralysis. For this reason, the use of *B. sempervirens* twigs as a snack can be dangerous. Toxic compounds attributed to *A. italicum* subsp. *italicum* are a coniine-like alkaloid (similar to the toxin in *Conium maculatum* L.), the saponine arin and cyanogenic glycosides. At low doses, the alkaloid bryonicine of *B. dioica* can cause dizziness, vomiting, convulsions, digestive problems, and kidney damage. In *T. baccata*, the red flesh portion of the aril is the only nontoxic part of the plant and corresponds also to the eaten part. *Ilex aquifolium* L., *Robinia pseudacacia, Solanum nigrum* L., and *Spartium junceum* L. have “a strong toxicity” [102] and were all mostly consumed cooked. The toxic component of the *I. aquifolium* fruit is a saponine which, at high concentration, causes hemolysis and alteration in the permeability of the gut mucosal membranes [103]. The bark of *R. pseudacacia,* whose sucking/chewing as a snack is documented in Tuscany [33], contains high levels of robinin, a lectin inducing neurological and gastrointestinal symptomatology [104]. The alkaloids cytisine and sparteine and the glycoside scoparin make *S. junceum* flowers toxic enough to advise against their use in food. However, their use has been documented, to a small extent, in complex mixtures of herbal teas in Lebanon and Syria known as Zhourat [105,106]. For 92% of the 49 plants listed as “toxic” in Günthardt et al. [102] the toxic and the consumed parts were the same; 25 of them were consumed cooked, 8 raw, and 12 both cooked and raw. Finally, 110 species have a “weak toxicity” [102]; for 79% of them, a correspondence between the toxic and eaten part was observed. Out of the species categorized as “weak toxic”, there are some plants that should be eaten with some precaution because their long-term consumption at low doses can imply chronic toxicity. In particular, there is a growing concern about the cancer risks related to repeated exposure to pyrrolizidine alkaloids (PAs) [107]; these secondary metabolites are present in genera as *Borago*, *Echium*, *Pulmonaria*, *Symphytum* (Lycopsamine-type pyrrolizidine alkaloids), *Jacobaea*, *Leucanthemum, Petasites*, and *Senecio* (Senecionine-type pyrrolizidine alkaloids). Due to their content of PAs, the use of these plants as components of herbal medicines is increasingly seen as a public health issue and it is subjected to regulatory recommendations and restrictions in many countries [108]. No information exists on the regulation of their food uses in Italy or in other countries. Estragole is a phenylpropanoid present in *Foeniculum vulgare*; the genotoxic and hepatocarcinogenic activity of estragole has been demonstrated both in vitro and in vivo [109]. Some plants are also rich in anti-nutritional factors: for example, the shoots of *Portulaca oleracea* L. and *Chenopodium album* subsp. *Album*, the epigeal parts of *Rumex* sp.pl., the rizhomes of *A. italicum* subsp. *Italicum*, and the leaves of *Amaranthus retroflexus* L. and *Silene vulgaris* contain high rates of calcium oxalate, possibly increasing the risk of nephrolithiasis. Low oxalic acid content was observed in the leaves of *Cichorium intybus* and *Chondrilla juncea* L., in the shoots of *Humulus lupulus* L., and in the flowering parts of *Malva sylvestris* [14]; these plants should be preferred by people who are prone to the formation of kidney stones.

High accumulation of nitrate occurs in many species (e.g., *C. intybus*, *Papaver rhoeas* subsp. *Rhoeas*, *P. oleracea*, *Sinapis arvensis*, and *Urtica dioica* subsp. *Dioica*) [110]. Health concerns are related to the capacity of nitrites, originating from nitrates, to produce nitrosamines, which are a risk factor for stomach cancer [111]. Genotypic, environmental, and developmental factors can affect the content of these anti-nutritional factors [99,112]. Moreover, cooking or heat treatments can drastically reduce their content and the associated health risks [87].

Many wild vegetable plants are able to grow in environments strongly affected by humans and potentially exposed to high levels of heavy metal pollution, such as roadsides, agricultural areas, abandoned fields, and urban areas. A regional analysis of Tuscan vineyards showed, for example, a rather high mean copper concentration (64.81 mg kg^−1^) [113], probably due to the large use of copper-based products in agriculture [114]. High mercury content has been found in some areas of Tuscany [115], due to pollution coming from geothermal systems and from mining activity. In the area of Monte Amiata, for example, is located the third-highest mercury deposit of Europe [116]. Based on the assessment of areas at risk of soil contamination in Europe, Tóth et al. [117] included Central Italy among the European regions that should be monitored more in detail. Moreover, hyperaccumulation is common in some wild food species such as *Hypochaeris radicata* L. [118] *Malva sylvestris* [119], *Plantago* sp.pl. [120], *R. psudoacacia* [121], *Rumex obtusifolius* L. [122], and *Taraxacum* sect. *Taraxacum* [123]. Accumulation of heavy metals in the human body via the food chain is believed to cause gastrointestinal cancer, malfunctioning of the immunological system, renal and skeletal damage, and neurological disorders [124]. Heavy metals also have negative impacts on the production [125,126] and nutritional properties of food plants [127]; in general, plants growing on metal-polluted soils show a deficiency in macro- and micronutrients, including lipids, proteins, vitamins, calcium, iron, and zinc.

In addition, the contact of wild food plants with the feces of livestock or wildlife hosting parasites and pathogen enterobacteria can be an important health risk [128,129,130].

### 3.10. Contribution of Wild Food Plants as Food Medicines

A total of 211 food species have traditional medicinal uses, and represent an important part of the regional medicinal ethnoflora. Most of them (113) are used to treat digestive system diseases, 48 for respiratory system diseases, and 47 for urinary tract diseases. Of the 211 taxa, 89% are ingested in specific preparations (e.g., decoctions, infusions, macerate, syrups) and 51 are used directly as food (cooked vegetables or salads) for their anti-inflammatory, depurative, emollient, remineralizing and vitaminizing properties. Examples of the latter plants are *Bellis perennis*, *Blitum bonus-henricus* and *Crepis* sp.pl. (cooked leaves as regulator of intestinal functions), *Taraxacum* sect. *Taraxacum* (cooked and raw leaves eaten as depurative, diuretic, stomachic, and cholagogue), and *Urtica dioica* subsp. *dioica* (raw or cooked leaves as anti-inflammatory of the urinary tract and intestinal astringent). For 168 species the same plant part served both as food and as medicine. A connection between food and medicine has been well known for a long time, both in books of ancient medicine and in the orally transmitted practices of popular medicine. This border is even more blurry in wild plants than in crops, because secondary compounds responsible for medicinal properties have not been subjected to the strong human selection involved in the domestication process [131]. Referring to *B. perennis*, Matthioli [132] writes: “the fresh herb eaten in salad softens the constipated intestine, and the same it makes when eaten cooked” or referring to *Cichorium intybus*: “the plant cooked and eaten comforts the stomach; eaten raw heals the dysentery…when cooked in the vinegar mitigates the urinary pains”. In the past, leafy vegetables collected at the beginning of the spring were perceived by people as “blood clearing” and “good for the liver”, a depurative and refreshing food able to clean the body from the carbohydrates and fats accumulated during the winter diet and to fight inflammation. Depurative and emollient action is due to the presence of mucilage made up of complex polysaccharide having decongestive, demulcent, diuretic, and laxative properties. Some examples of plants having a high mucilage content are *Malva sylvestris* [133], *B. perennis*, and *Plantago major* L. [134].

The use of seasoning plants in gastronomy and their beneficial effects on health have been widely investigated. In particular, they are recognized to have a digestive and carminative action when added to high-fat foods. The medicinal importance of aromatic plants is marked by the fact that 44 species recorded in this study are also used in the local ethnopharmacology to treat digestive problems. As observed by Pieroni [135], some of these plants are also used in the form of decoction or infusion at the end of the meal with similar aims. Moreover, essential oils extracted from *Mentha* sp.pl., *Salvia rosmarinus* Schleid., and *Thymus* sp.pl. have strong antibacterial and antifungal properties which help to fight foodborne microorganisms and to reduce food poisoning [136].

### 3.11. Wild Food Plants for Agriculture

Some wild food plants are crop ancestors, often of vegetables. Wild and domesticated plants are sometimes taxonomically indistinguishable at the specific level, although they differ genetically due to a strong selection pressure during domestication. One emblematic example is *Cichorium intybus*, an herbaceous perennial with several cultivated forms used for different purposes (leafy vegetable, root vegetable, Witloof salad, coffee substitute, inulin production) and subjected to different selection rates [137]. Lettuce, the most popular salad crop worldwide with many types different in shape, color, texture, and taste [138], belongs to the species *Lactuca sativa* L., which is found in the wild as *Lactuca sativa* L. subsp. *serriola* Galasso, Banfi, Bartolucci, and Ardenghi [139]. Other examples of major vegetable crop species recorded in Tuscany as wild are fennel (*Foeniculum vulgare*), carrot (*Daucus carota* L.), and cardoon (*Cynara cardunculus* L. subsp. *cardunculus*). For these species, the wild relatives can still represent a genetic resource for breeding as the selective approach during domestication may have left behind valuable alleles (e.g., genes for biotic and abiotic stress resistance) [139]. For other wild food species, the genetic impact of cultivation has been less pronounced [140]. This is the case of aromatic plants (e.g., sage, *Salvia officinalis* L.; rosemary, *Salvia rosmarinus*; thyme, *Thymus* sp.pl.) and minor vegetables, grown on small areas and often intended for local markets, or cultivated by amateur growers. The seeds can be supplied by specialized seed companies, and be more or less selected, or even collected in the wild. Some examples are borage (*Borago officinalis*), horseradish (*Armoracia rusticana* G.Gaertn., B.Mey., and Scherb.), dandelion (*Taraxacum* sect. *Taraxacum*), sorrel (*Rumex acetosa*), and nettle (*Urtica dioica*) [141]. Finally, many other wild food plants, still uncultivated, could be considered worthy of study to begin their cultivation/domestication [130]. Domestication is a complex process, driven by genetic, agronomic, and cultural changes and their interactions [142]. Ethnobotanical knowledge can certainly contribute to this process. In fact, when evaluating the suitability for domestication of a possible candidate plant, it is necessary to consider the human relationship with it [142].

A good tolerance to different kinds of stress (e.g., drought, salinity, high temperature, nutrient shortage) (see Section 3.3) and a high nutritional value (see Section 3.8) are common traits of wild food plants, making them promising candidates as new crops. Furthermore, their abundance in secondary metabolites with antioxidant and healthy properties can meet consumer demand for functional foods. Cultivating wild vegetables is also a way to promote and preserve the ethnobotanical heritage of an area with its biological and cultural components, including the protection of genetic diversity from threats such as unsustainable gathering practices and habitat degradation [110,143]. At the same time, it results in an increased crop diversification, producing new opportunities for the growers and benefits for the consumers in terms of a richer and healthier diet. It cannot be overlooked, however, that the wild-collected plants may differ from the cultivated counterparts in chemical composition, and therefore in their nutraceutical properties as well as in their sensory profile [99]. For example, in *Picris hieracioides*, *Poterium* *sanguisorba*, and *Plantago coronopus*, different cultivation systems resulted in a decrease in their antioxidant activity, while in *C. intybus*, the plants cultivated in the open air and in a soilless system showed an antioxidant activity close to that of the plants collected in the wild. The sensory profile of soilless-cultivated *P. hieracioides* and *C. intybus* was the most different compared with that of the wild-collected plants [99].

The cultivation of wild food plants could reduce the health risks that the consumption of gathered specimens may cause due to possible pollutant accumulation and biological contamination (see Section 3.10). Growing wild crops could also be advantageous in reducing the risks of foodborne outbreaks, due to a lower susceptibility to microbiological contamination in comparison with the intensively domesticated genotypes [144].

Wild leafy greens, a plant category frequently recorded in the edible Tuscan flora (see Section 3.5), may contribute to the development of emerging horticultural sectors such as vertical farming and microgreens production. Vertical farming (the soilless cultivation of crops in multiple levels of horizontal growing platforms or in vertical surfaces [145]), currently focuses on salads due to their small size, high value, rapid growing, and small footprint [146]. Microgreens are small salad greens consisting of seedlings harvested without roots within 10–20 days from seedling emergence, when cotyledons are fully expanded, and the first pair of true leaves are more or less developed [147]. Since their entrance in the market in the 1980s in the US, they have been gaining more and more popularity as a specialty culinary ingredient used to enhance the color, taste, texture, and nutrient value of many dishes [148]. This makes the availability of a wide range of species very appreciated, and the wild greens excellent candidates to enlarge such range of produce. Some wild greens, including species recorded in this work (*P. sanguisorba, Sinapis arvensis*, *Taraxacum* sect. *Taraxacum* [98]; *R. acetosa, P. coronopus, Portulaca oleracea* [149]) have been recently tested for microgreen production with promising results.

## 4. Conclusions

The traditional Tuscan landscape consists of a perfect balance of nature and culture as a result of environmental factors and human actions. Data presented and discussed in this paper provide information on the use of wild food plants in the Tuscan gastronomic tradition, and show that these biological resources can play an important role even today. Food, liquor, and/or seasoning uses of 357 taxa (3711 URs) were recorded in the region. This number of taxa, including 12 species not previously reported in literature concerning Tuscan ethnobotany, is higher than that of other Italian regions, such as Apulia and Sicily, the object of many ethnobotanical studies, recently reviewed. Considering the whole of Italy, 52 taxa over the total recorded in Tuscany seem to be exclusive of this region. These figures, as well as the versatility observed in the use of many species in terms of both utilized plant parts and recipes they are intended for, demonstrate the richness of traditional Tuscan ethnobotany. Such biocultural heritage, on one hand, needs to be safeguarded from the risks of progressive depletion related to the ongoing disappearance of the rural society; on the other hand, it can be a “source of inspiration”, in the light of current scientific and technical knowledge, for different fields of human activity, including agriculture. Wild edible plants can be a genetic resource of valuable genes for breeding as well as a reservoir of potential new crops. Wild Tuscan flora, with 219 species used as leafy greens, could offer interesting perspectives of exploitation for emergent agricultural sectors such as vertical farming and microgreens production. Moreover, the nutraceutical value of many wild food plants could meet consumer demand for functional foods. Current research still appears to be focused only on a few species, some of which have been already introduced in cultivation (e.g., *Atriplex hortensis*, *Cichorium intybus*, *Portulaca oleracea*, *Taraxacum* sect. *Taraxacum, Urtica dioica* subsp. *dioica*). Further research is needed to assess the agronomic potential of other wild species listed in this paper. The future studies should be also oriented on the aspect of nutritional and healthy properties of these plants.

Finally, it should also be recalled that the use of wild plants is closely linked to the environment and its preservation. In this regard, knowing and passing on the local traditions is a way to maintain that specific link existing between the communities and the surrounding environment, where traditional knowledge has been formed and experienced.

## Figures and Tables

**Table 1:** List of the 30 most cited taxa in Tuscany.

Species	Family	Origin/Alien Status	Used Parts	Use	Preparation	Recipes	#Records	URs	CI_s_	Italian Regions	European Countries
*Foeniculum vulgare* Mill.	Apiaceae	N	Br, Ep, Fl, Fr, Hy, Le, Se, Sh, St	F, S	R, C	beverage, cooked vegetables, omelets, salads, snacks, sweets	51	68	1.015	C, ABR, BAS, CAL, CAM, EMI, LAZ, LIG, MAR, MOL, PUG, SAR, SIC, UMB	AR, BA, CY, ES, GE, HR, PT
*Taraxacum* FH.Wigg. sect. *Taraxacum*	Asteraceae	N	Ep, Fl, Hy, Le, Wh	F	R, C	coffee substitute, cooked vegetables, omelets, pickles, quiche filling, ravioli filling, risotto, salads	51	81	1.209	C, ABR, BAS, CAL, CAM, EMI, FRI, LAZ, LIG, LOM, MAR, PIE, PUG, SAR, SIC, TRE, UMB, VEN	AL, AR, BA, BG, BY, CH, EE, ES, GE, HR, MK, NL, PL, RO, RS, RU, SK, UA, XK
*Borago officinalis* L.	Boraginaceae	N	Ep, Fl, Le	F	R, C	cooked vegetables, fried, omelets, pasta/dumplings, quiche filling, ravioli filling, risotto, salads	49	106	1.582	C, BAS, CAL, CAM, EMI, LIG, LOM, MAR, MOL, PIE, PUG, SAR, SIC, UMB	BA, CH, CY, ES, HR, PT
*Cichorium intybus* L.	Asteraceae	N	Hy, Le, Sh, Wh	F	R, C	coffee substitute, cooked vegetables, omelets, quiche filling, ravioli filling, salads, snacks	49	79	1.179	C, ABR, BAS, CAL, CAM, EMI, FRI, LAZ, LIG, LOM, MAR, MOL, PUG, SAR, SIC, UMB	AR, BA, CH, CZ, CY, EE, ES, HR, NL, PL, RS, RU, SK, UA
*Sonchus oleraceus* L.	Asteraceae	N	Ep, Hy, Le	F	R, C	coffee substitute, cooked vegetables, omelets, quiche filling, ravioli filling, salads	44	70	1.045	C, ABR, BAS, CAL, CAM, LIG, MAR, MOL, PUG, SAR, SIC, UMB, VEN	BA, CH, CY, CZ, EE, ES, HR, PT, SK, XK
*Clinopodium nepeta* (L.) Kuntze	Lamiaceae	N	Br, Ep, Fl, Le, Wh	L, S			43	45	0.672	C, CAL, CAM, EMI, MAR, PUG, SIC, UMB	ES, HR, MK, PT
*Papaver rhoeas* L. subsp. *rhoeas*	Papaveraceae	N	Ep, Fl, Le, Se	F, S	R, C	cooked vegetables, jam, omelets, quiche filling, ravioli filling, salads	40	65	0.970	C, BAS, CAL, CAM, EMI, LIG, MAR, MOL, PIE, PUG, SAR, SIC, TRE, UMB, VEN	BA, BG, CY, CZ, ES, HR, PL, PT, RO, SK
*Poterium sanguisorba* L.	Rosaceae	N	Ep, Le, Wh	F, S	R, C	cooked vegetables, omelets, quiche filling, ravioli filling, salads	36	48	0.716	C, CAM, EMI, LIG, MAR, PIE, PUG, SIC, UMB	BA, CH, ES, HR, SK
*Reichardia picroides* (L.) Roth	Asteraceae	N	Ep, Le, Sh	F	R, C	cooked vegetables, omelets, ravioli filling, quiche filling, salads	36	54	0.806	C, ABR, BAS, CAL, CAM, LIG, MAR, PUG, SAR, SIC, UMB	BA, ES, HR
*Campanula rapunculus* L.	Campanulaceae	N	Ep, Hy, Le, Wh	F	R, C	cooked vegetables, ravioli filling, quiche filling, salads	35	42	0.627	C, LIG, LOM, MAR, PUG, UMB, VEN	BA, CH, CZ, ES, PT
*Clematis vitalba* L.	Ranunculaceae	N	Ep, Le, Sh	F	R, C	cooked vegetables, omelets, pasta/dumplings, quiche filling, ravioli filling, risotto, salads, snacks, sweets	33	62	0.925	C, ABR, BAS, CAL, CAM, EMI, LAZ, LIG, MAR, MOL, PUG, SIC, UMB	BA, ES, HR, MK, SK
*Helminthotheca echioides* (L.) Holub	Asteraceae	N	Le, Wh	F	R, C	cooked vegetables, quiche filling, ravioli filling, salads	33	41	0.612	C, BAS, CAL, CAM, LIG, MAR, MOL, PUG, SIC, UMB, VEN	BA, ES, HR
*Laurus nobilis* L.	Lauraceae	N	Fr, Le, Sh	F, L, S	R	snacks	32	32	0.478	ABR, BAS, CAL, CAM, EMI, FRI, LAZ, LIG, LOM, MAR, MOL, PUG, SAR, SIC, VEN	AL, BA, BG, CY, ES, GE, HR
*Urtica dioica* L. subsp. *dioica*	Urticaceae	N	Ep, Fl, Hy, Le, Sh, Wh	F	R, C	cooked vegetables, fried, omelets, pasta/dumplings, quiche filling, ravioli filling, risotto, salads	41	97	1.448	C, ABR, BAS, CAL, CAM, EMI, FRI, LAZ, LIG, LOM, MAR, MOL, PIE, PUG, SAR, SIC, TRE, UMB, VEN	AL, AR, BA, BG, BY, CH, CZ, EE, ES, GE, HR, MK, NL, PL, PT, RO, RS, RU, SK, UA, XK
*Silene vulgaris* (Moench) Garcke	Caryophyllaceae	N	Ep, Fl, Le, Sh	F	R, C	cooked vegetables, omelets, pasta/dumplings, quiche filling, ravioli filling, risotto, salads	32	77	1.149	C, ABR, CAL, CAM, EMI, FRI, LIG, LOM, MAR, PIE, PUG, SAR, SIC, TRE, UMB, VEN	BA, BY, CY, CZ, ES, HR, PL
*Hypochoeris radicata* L.	Asteraceae	N	Ep, Le, Wh	F	R, C	cooked vegetables, omelets, risotto, salads	30	41	0.612	C, CAL, CAM, LIG, PUG, SAR, SIC, UMB, VEN	BA, ES, HR
*Origanum vulgare* L. subsp. *vulgare*	Lamiaceae	N	Br, Fl, Le	S			30	30	0.448	ABR, BAS, CAL, CAM, FRI, LAZ, LIG, LOM, MAR, MOL, PUG, SIC, UMB	AL, AR, BA, BG, BY, CH, CZ, EE, ES, HR, MK, PL, PT, RO, RS, SK, UA, XK
*Plantago lanceolata* L.	Plantaginaceae	N	Ep, Le	F	R, C	cooked vegetables, pasta/dumplings, quiche filling, salads	29	37	0.552	BAS, CAM, EMI, FRI, LIG, MAR, PIE, PUG, SIC, UMB, VAL, VEN	AR, AT, BA, BY, CH, CZ, ES, HR, NL, PL
*Asparagus acutifolius* L.	Asparagaceae	N	Sh	F	C	cooked vegetables, omelets, pasta/dumplings, quiche filling, risotto	28	55	0.821	C, ABR, BAS, CAL, CAM, EMI, FRI, LAZ, LIG, LOM, MAR, MOL, PIE, PUG, SAR, SIC, UMB	BA, CY, ES, HR
*Sambucus nigra* L.	Viburnaceae	N	Fl, Fr, Le	F, L, S	R, C	beverage, cooked fruits, fried, jam, omelets, salads, sweets	28	63	0.940	C, BAS, CAL, CAM, EMI, FRI, LIG, LOM, MAR, MOL, PIE, SAR, SIC, TRE, VEN	AT, BA, BG, BY, CH, CZ, EE, ES, HR, HU, MK, NL, PL, RO, RS, SK, UA, XK
*Daucus carota* L.	Apiaceae	N	Ep, Fl, Fr, Hy, Le	F	R, C	beverage, cooked vegetables, omelets, salads, snacks	26	40	0.597	BAS, CAL, CAM, LIG, LOM, MAR, PUG, SAR, SIC, UMB	AL, AR, BA, BG, CZ, EE, ES, GE, HR, MK, NL, PL, PT, SK, UA, XK
*Robinia pseudacacia* L.	Fabaceae	A/ Ne Inv	Ba, Fl, Sh	F, L	R, C	beverage, cooked vegetables, fried, jam, snacks	25	31	0.463	C, BAS, CAL, CAM, EMI, FRI, LIG, MAR, MOL, PIE, PUG, SAR	BA, BY, CH, CZ, ES, GE, HR, HU, PL, RO, SK, UA
*Thymus longicaulis* C. Presl subsp. *longicaulis*	Lamiaceae	N	Br, Ep, Fl, Le	F, S	C	beverage	24	25	0.373	ABR, BAS, CAM, FRI, LAZ, LIG, LOM, MOL, UMB	AL, BG, BY, EE, ES, HR, MK, PL, RO, RU, UA, XK
*Ficaria verna* Huds.	Ranunculaceae	N	Fl, Hy, Le, Wh	F	R, C	cooked vegetables, omelets, picklets, quiche filling, salads	23	32	0.478	CAL, LIG, PIE, SAR, SIC, UMB	EE, HR, PL, RO, SK
*Humulus lupulus* L.	Cannabaceae	N	Le, Sh	F, L	C	cooked vegetables, omelets, quiche filling, risotto	23	33	0.493	BAS, CAL, CAM, EMI, FRI, LAZ, LIG, LOM, MAR, MOL, PIE, PUG, SAR, TRE, UMB, VAL, VEN	AR, BA, BG, BY, CZ, EE, ES, HR, HU, PL, PT, RO, SK, UA
*Rosa canina* L.	Rosaceae	N	Fl, Fr, Sh	F, L	R, C	beverage, cooked vegetables, flour substitute, jam, omelets, raw fruits, salads, snacks	23	48	0.716	ABR, BAS, CAL, CAM, EMI, FRI, LAZ, LIG, LOM, MAR, PIE, PUG, SAR, SIC, TRE	AL, AR, BA, BG, BY, CH, CZ, EE, ES, HR, HU, MK, PL, RO, RS, RU, SK, UA, XK
*Bellis perennis* L.	Asteraceae	N	Fl, Le, Wh	F	R, C	beverage, cooked vegetables, omelets, quiche filling, salads	22	33	0.493	C, ABR, CAM, EMI, LIG, MAR, PIE, PUG, SIC, UMB	AT, BA, BG, CH, CZ, ES, HR, MK, NL, PL
*Malva sylvestris* L.	Malvaceae	N	Ep, Fl, Hy, Le, Sh	F	R, C	cooked vegetables, pasta/dumplings, quiche filling, ravioli filling, risotto, salads	22	35	0.522	BAS, CAL, CAM, FRI, LAZ, LIG, LOM, LOM, MAR, PIE, PUG, SAR, SIC, UMB, VAL, VEN	AL, AR, BA, BG, CY, CZ, ES, GE, HR, MK, PL, PT, RO, SK, XK
*Rubus ulmifolius* Schott	Rosaceae	N	Fr, Sh	F, L	R, C	beverage, cooked fruits, cooked vegetables, jam, omelets, quiche filling, raw fruits, snacks, sweets	22	57	0.851	C, ABR, BAS, CAL, CAM, LIG, MAR, PUG, SAR, SIC, UMB	AL, BA, ES, HR, PT
*Rumex crispus* L.	Polygonaceae	N	Le	F	R, C	cooked vegetables, omelets, ravioli filling, quiche filling, salads	22	25	0.373	ABR, CAM, LIG, PUG, SAR, SIC, UMB	AR, BA, BY, CZ, EE, ES, HR, PL, SK

**Origin:** N = native, A = alien; **Alien status:** Ar = archaeophyte, Ne = neophyte, Nat = naturalized, Inv = invasive; **Used parts:** Ba = bark, Br = branches, Ep = epigeal organs, Fl = flowers, Fr = fruits, Le = leaves, Hy = hypogeal organs, Se = seeds, Sh = shoots, St = stems, Wh = whole plant; **Use:** F = food, L = liquor, S = seasoning; **Preparation:** R = raw, C = cooked; **#Records** = number of records; **Italian Regions**: C = common to many regions, ABR = Abruzzo, BAS = Basilicata, CAL = Calabria, CAM = Campania, EMI = Emilia-Romagna, FRI = Friuli-Venezia Giulia, LAZ = Lazio, LIG = Liguria, LOM = Lombardy, MAR = The Marches, MOL = Molise, PIE = Piedmont, PUG = Apulia, SAR = Sardinia, SIC = Sicily, TRE = Trentino-Alto Adige, UMB = Umbria, VAL = Aosta Valley, VEN = Veneto; **European Countries**: AL = Albania, AR = Armenia, AT = Austria, BY = Belarus, BA = Bosnia–Herzegovina, BG = Bulgaria, HR = Croatia, CY = Cyprus, CZ = Czech Republic, EE = Estonia, GE = Georgia, HU = Hungary, XK = Kosovo, MK = Macedonia, NL = Netherlands, PL = Poland, PT = Portugal, RO = Romania, RU = Russia, RS = Serbia, SK = Slovakia, ES = Spain, CH = Switzerland, UA = Ukraine.

**Table 2:** Species showing the best adaptation to four major stress factors: T (temperature, range 1–12), U (edaphic humidity, range 1–11), N (nutrients, range 1–9), and S (salinity, range 0–3). u = unknown.

	T	U	N	S
Taxa well adapted to heat and nutrient-poor soils				
* Rumex bucephalopharus*	12	2	1	0
Taxa well adapted to drought and nutrient-poor and saline soils				
* Crithmum maritimum*	8	1	1	3
Taxa well adapted to drought and saline soils				
* Atriplex halimus*	10	1	2	3
Taxa well adapted to nutrient-poor soils				
* Brassica oleracea*	10	2	1	1
* Capparis spinosa*	10	2	1	1
* Andryala integrifolia*	9	2	1	0
* Valerianella eriocarpa*	9	2	1	0
* Centranthus ruber*	8	2	1	0
* Echinophora spinosa*	8	4	1	1
* Hyoseris radiata*	8	2	1	0
* Hypochoeris radicata*	8	2	1	0
* Teucrium polium*	8	2	1	0
* Thymus striatus* subsp. *acicularis*	8	2	1	0
* Thymus vulgaris*	8	2	1	0
* Tolpis virgata*	8	2	1	0
* Salvia officinalis*	6	2	1	0
* Teucrium chamaedrys*	6	2	1	0
* Vaccinium uliginosum* subsp. *uliginosum*	4	9	1	0
* Sedum album*	u	2	1	0
* Thymus pulegioides*	u	4	1	0
Taxa well adapted to dry soils				
* Teucrium montanum*	7	1	2	0
Taxa well adapted to saline soils				
* Salicornia perennans* subsp. *perennans*	7	8	7	3

**Table 3:** Diversity indices calculated for the categories of use and for each food preparation. #Taxa = number of taxa listed; URs = number of use reports; Simpson: Simpson index of diversity (1-D); Shannon H = Shannon diversity index; Chao1: unbiased Chao1 richness.

Uses	#Taxa	URs	Simpson	Shannon H	Chao1
Food	324	3115	0.98	5.05	414.30
Liquor	49	178	0.97	3.71	60.93
Seasoning	81	418	0.96	3.67	149.70
Detailed food uses					
Beverage	28	55	0.94	3.23	61.58
Cooked vegetables	197	1190	0.98	4.63	309.40
Cooked fruits	19	33	0.91	2.84	52.94
Coffee substitute	12	30	0.91	2.45	14.42
Flour substitute	14	25	0.87	2.50	40.40
Fried	25	84	0.86	2.56	58.60
Jam	44	211	0.96	3.15	63.64
Omelets	73	231	0.95	3.73	130.20
Pasta/dumplings	13	29	0.90	2.51	19.76
Pickles	14	25	0.90	2.60	28.40
Quiche	78	127	0.98	4.42	158.40
Ravioli	53	112	0.95	3.66	199.90
Raw fruits	41	184	0.96	3.48	49.21
Risotto	24	47	0.94	3.07	68.37
Salads	148	540	0.98	4.55	246.10
Snacks	61	145	0.97	3.99	102.20
Sweets	25	46	0.97	3.34	32.17

**Table 4:** First five top-ranked taxa for the most important macro- and micronutrients.

Nutrients	Taxa	Plant Part	Content	Reference
			(g or mg /100 g FW)	
Proteins	*Pinus pinea*	seeds	32.0	[84]
	*Fagus sylvatica*	seeds	22.0	[84]
	*Juglans regia*	seeds	20.0	[84]
	*Atriplex hortensis*	leaves	17.0	[90]
	*Corylus avellana*	seeds	15.0	[84]
Carbohydrates	*Ceratonia siliqua*	fruits	80.0	[84]
	*Atriplex hortensis*	leaves	56.0	[90]
	*Castanea sativa*	fruits	42.75	[91]
	*Rosa canina*	fruits	38.42	[92]
	*Mentha pulegium*	leaves	34.40	[14]
Lipids	*Corylus avellana*	fruits	62.0	[84]
	*Juglans regia*	seeds	60.0	[84]
	*Fagus sylvatica*	fruits	50.0	[84]
	*Pinus pinea*	seeds	48.0	[84]
	*Hippophaë fluviatilis*	fruits	7.0	[84]
Ca	*Atriplex hortensis*	leaves	*2000*	[90]
	*Urtica dioica*	leaves	*625*	[13]
	*Sisymbrium officinale*	leaves	*495*	[84]
	*Bellis perennis*	leaves	*444*	[64]
	*Bunias erucago*	leaves	*425*	[64]
P	*Pinus pinea*	seeds	*508*	[84]
	*Juglans regia*	seeds	*380*	[84]
	*Corylus avellana*	fruits	*290*	[84]
	*Atriplex hortensis*	leaves	*150*	[90]
	*Sisymbrium officinale*	leaves	*125*	[84]
Fe	*Bellis perennis*	leaves	*40.80*	[64]
	*Punica granatum*	seeds	*32.30*	[93]
	*Bunias erucago*	leaves	*24.10*	[64]
	*Amaranthus retroflexus*	shoots	*12.34*	[94]
	*Atriplex hortensis*	leaves	*10.0*	[90]
Na	*Crithmum maritimum*	leaves	464	[14]
	*Cakile maritima*	leaves	*308*	[83]
	*Helminthotheca echioides*	leaves	*184*	[95]
	*Sonchus oleraceus*	leaves	*144*	[14]
	*Sonchus asper*	leaves	*137*	[14]
K	*Bunias erucago*	leaves	*2200*	[64]
	*Bellis perennis*	leaves	*2053*	[64]
	*Chenopodium album*	leaves	*1155*	[14]
	*Fagus sylvatica*	fruits	*1018*	[84]
	*Chondrilla juncea*	leaves	*1015*	[14]
Mg	*Punica granatum*	seeds	*1697*	[93]
	*Atriplex hortensis*	leaves	*500*	[90]
	*Corylus avellana*	fruits	*284*	[84]
	*Malva sylvestris*	leaves	*283*	[14]
	*Poterium sanguisorba*	leaves	*282*	[64]
Mn	*Capparis spinosa*	flowers	*25.90*	[96]
	*Atriplex hortensis*	leaves	*10.0*	[90]
	*Rubus ulmifolius*	fruits	*15.60*	[14]
	*Malva sylvestris*	leaves	*498*	[14]
	*Urtica dioica*	leaves	*1.70*	[14]
Zn	*Punica granatum*	seeds	*5.81*	[93]
	*Chondrilla juncea*	leaves	*1.63*	[14]
	*Malva sylvestris*	shoots	*1.58*	[14]
	*Asparagus acutifolius*	shoots	*1.06*	[14]
	*Chenopodium album*	leaves	*1.03*	[14]
Cu	*Punica granatum*	seeds	*6.82*	[93]
	*Atriplex hortensis*	leaves	*2.0*	[90]
	*Arbutus unedo*	fruits	*0.99*	[14]
	*Silybum marianum*	leaves	*0.80*	[14]
	*Chondrilla juncea*	leaves	*0.43*	[14]
Vit. C	*Hippophaë fluviatilis*	fruits	*450*	[84]
	*Rosa canina*	fruits	*426*	[92]
	*Primula veris*	leaves	*418*	[3]
	*Primula vulgaris*	leaves	*305*	[3]
	*Urtica dioica*	leaves	*285*	[14]

## Data Availability

The data presented in this study are available on request from the corresponding authors.

## Supplementary Materials

The following supporting information can be downloaded at: https://www.mdpi.com/article/10.3390/foods11030300/s1, Supplementary material S1: Consulted ethnobotanical literature on the use of wild food plants in Tuscany, Italy, and Europe; Supplementary Material S2: Wild food plants consumed in Tuscany.

## Abbreviations

CIs	Cultural Importance Index for species
CIu	Cultural Importance Index for use categories
EI	Portéres Ethnobotanicity Index
H	Shannon Information or Uncertainty index
PAs	pyrrolizidine alkaloids
