# Supplementary material for: The Renaissance of Wild Food Plants: Insights from Tuscany (Italy)
Source: Foods. 2022 Jan 23;11(3):300. doi: 10.3390/foods11030300 (PMC8834290; doi:10.3390/foods11030300)
Supplement: Supplementary file 1 [file foods-11-00300-s001.zip › foods-1564857-supplementary/Supplementary material S1 rev.pdf]

Supplementary material S1

List of consulted bibliographic sources

Tuscany

1. Corsi, G.; Gasparri, G.; Pagni, A.M. L'uso delle piante nell'economia domestica della Versilia collinare e montana. *Atti Soc. Tosc. Sci. Nat., Memorie, Serie B*, **1981**, *87,* 309-386.
2. Corsi, G.; Gasparri, G.; Pagni, A.M. Studi sulla flora e vegetazione del monte pisano (Toscana Nord occidentale). Le piante spontanee nell'alimentazione popolare. *Atti Soc. Tosc. Sci. Nat., Memorie, Serie B*, **1979**, *86,* 79-101.
3. Gori, D. Indagine etnobotanica nella Valle del Santerno: saperi tradizionali sulle piante nel territorio di Firenzuola (FI). Master's Degree thesis, University of Florence, 2008.
4. Randellini, L. L'uso delle piante nella tradizione popolare dell'alto Casentino (AR). Master's Degree thesis, University of Florence, 2007.
5. Signorini, M.A.; Lombardini, C.; Bruschi, P.; Vivona, L. Conoscenze etnobotaniche e saperi tradizionali nel territorio di San Miniato (Pisa). *Atti Soc. Tosc. Sci. Nat., Menorie Serie B,* **2007***, 114,* 65-83.
6. Frassinelli, N. Indagine sulle conoscenze etnobotaniche a S. Agata (Scarperia, FI). Bachelor Degree thesis, University of Florence, 2008.
7. Frondizzi, F. "La natura non fa nulla d'inutile" - Le piante e gli usi popolari: indagine etnobotanica nel comune di Pontassieve (Firenze). Bachelor Degree thesis, University of Florence, 2009.
8. Lenzarini, C. Gli usi tradizionali delle piante: indagine etnobotanica nel Comune di Vaglia (Firenze). Bachelor Degree thesis, University of Florence, 2006.
9. Egea, T.; Signorini, M.A.; Ongaro, L.; Rivera, D.; Obón de Castro, C.; Bruschi, P. Traditional alcoholic beverages and their value in the local culture of the Alta Valle del Reno, a mountain borderland between Tuscany and Emilia-Romagna (Italy). *J. Ethnobiol. Ethnomed.* **2016**, *12*(1), 27.
10. Giachetti, G.; Tomei, P.E. Piante officinali nell'uso tradizionale del territorio mugellano (Toscana). *Atti Soc. It. Sci. Nat. and Museo Civico Storia Naturale*, Milano, **2003**, 144, 23-49.
11. Tomei, P.E.; Trimarchi, S. *Piante d'uso etnobotanico in Toscana*. Pacini-Fazzi Editore: Lucca, Italy, 2017.
12. Tomei, P.E.; Lippi, A.; Uncini Manganelli, R.E. *Funghi Tartufi ed Erbe mangerecce.* Atti del Convegno internazionale di studi, L'Aquila (september 28-1st october 1995). Università degli Studi de L'Aquila & Accademia Italiana della Cucina, 1996.
13. Pieroni, A. "Erbi boni" nelle tradizioni gastronomiche garfagnine. In Erbi boni, erbi degli streghi/Good weeds, witches' weeds. Atti del seminario di studio, Gallicano (LU) (May 27 1998) Köln, Germany: experiences Verlag, 1998, 3-18.
14. Tomei, P.E.; Camangi, F. La cucina massese e carrarese: aspetti alimurgici e botanici. *Paralleli e Meridiani* **2004**, *2*(1): 55-66.
15. Ghirardini, M.P.; Carli, M.; Del Vecchio, N.; Rovati, A.; Cova, O.; Valigi, F.; Agnetti, G.; Macconi, M.; Adamo, D.; Traina, M. et al., The importance of a taste. A comparative study on wild food plant consumption in twenty-one local communities in Italy. *J. Ethnobiol. Ethnomed.* **2007**, *3*(1), 22.
16. Tomei, P.E.; Gaspari, G. Indagini sulle zone umide della Toscana. XVI. Le piante officinali dei bacini palustri della Toscana settentrionale. *Atti Soc. Tosc. Sci. Nat., Memorie, Serie D,* ***1981****, 88,* 175-194.

17. Della Monaca, G.; Roselli, D.; Tosi, G. La botanica popolare del Monte Argentario. In Arrigoni: *Geobotanica e Etnobotanica del Monte Argentario*; Arrigoni, P.V., Laurum Editrice, Pitigliano, Italy, 2001.
18. Pieroni, A. Piante spontanee della tradizione ed immaginario collettivo in Alta Garfagnana (Lucca): un centro di documentazione sulla cultura orale. *Inf. Bot. Ital.* 31, 183-189.
19. Benvenuti, R.; Covarelli, A.; Storai, R. Le erbe officinali del territorio della Comunità Montana Val di Bisenzio. Comunità Montana Val di Bisenzio, 1996.
20. Pieroni, A. Gathered wild food plants in the upper valley of the Serchio river (Garfagnana), central Italy. *Econ. Bot.* **1999**, *53(3),* 327–341.
21. Pieroni, A. Medicinal plants and food medicines in the folk traditions of the upper Lucca Province, Italy. *J. Ethnopharmacol.* **2000**, *70(3),* 235–273.
22. Tomei, P.E.; Camangi, F. Tradizioni alimurghiche in Toscana. Piante selvatiche e coltivate nella preparazione delle zuppe. Accademia Lucchese di Scienze, Lettere e Arti, 2014.
23. Camangi, F.; Uncini-Manganelli, R.E. Indagine etnobotanica nel Pistoiese: il territorio di Collodi. *Bull. Orto Bot. Lucca,* **2004**, *3(1),* 19-39.
24. Camangi, E.; Stefani, A.; Gianbastiani, M. Appunti fitoalimurgici: la “zuppa matta di Piegaio” (Val Pedogna-Lucca-Toscana). Comune di Lucca, Italy, 2005, pp 85-98.
25. Uncini-Manganelli, R.E.; Camangi, F.; Tomei, P.E. Primi appunti su alcune tradizioni alimentari nel territorio di Massaciuccoli (LU). In *Il Bacino del Massaciuccoli*; Pacini Editore, Pisa, Italy, 1999, 5, 49-53.
26. Camangi, F.; Stefani, A.; Tomei, P.E. Le tradizioni etnobotaniche nelle Foreste Casentinesi. Atti Convegno “Carlo Siemoni: selvicoltore granducale”, Poppi, Arezzo, Italy, 2003, pp. 71-85.
27. Camangi, F.; Uncini-Manganelli, R.E. L’etnobotanica nel territorio di Capannori: stato delle conoscenze e nuove acquisizioni. In *Aspetti biologici del territorio del Comune di Capannori. Studi Capannoresi*. Comune di Capannori, Lucca, Italy. La Grafica Pisana, Buti, Pisa, 1999, 3, 177-224.
28. Lippi, A. Da erbe per i poveri a cibo per i buongustai. La riscoperta delle piante selvatiche di uso alimentare. Ponte Eds.; Lucca, Italy, 1998.
29. Camangi, F.; Stefani, A.; Sebastiani, L. Piante e folclore nelle Valli di Zeri in Lunigiana. ETS Eds.; Pisa, 2013.
30. Pieroni, A. Toxic plants as food plants in the traditional uses of the Eastern Apuan Alps Region, North-West Tuscany, Italy. In *Il cibo e il corpo. Dal cibo alla cultura, dalla cultura al cibo/Food and body. From food to culture, from culture to food.* Erga Eds.; Genoa, Italy, 1999, 267-272.
31. Tomei, P.E.; Uncini Manganelli, R. E. Etnobotanica nel Padule di Fucecchio. In AA.VV. (Eds.), Iniziativa Comunitaria Equal, Aree Protette: Adattamento Professionale degli Occupati nel Comparto Agricolo: 181-194.
32. Belcari, M.S. Una ricerca etnobotanica nel territorio di S. Maria a Monte (Toscana): primo contributo. *Paralleli e meridiani*, 2012, 7-10, 7-47.
33. Camangi, F.; Stefani, A. L’orto dei semplici nell’eremo di Santa Caterina a Rio nell’Elba. ETS Eds.; Pisa, Italy, 2014.
34. Camangi, F.; Bettini, D.; Cecchelli, G.; Santoro, A.; Stefani, A. Piante spontanee d’uso alimentare. Viaggio alla scoperta della cucina povera a partire dalla tradizione popolare volterrana. II ed., ETS Eds.; Pisa, Italy, 2010.
35. Poli, I. Gli erbi boni della cucina tradizionale garfagnina. Tipografia Gasperetti, Fornaci di Barga, Lucca, Italy, 2012.

36. Camangi, F.; Uncini Manganelli, R.E. La cornucopia della natura: piante alimentari nella tradizione popolare del Monte Pisano. In *Monte Castellare – Valle delle Fonti: due aree protette dei Monti Pisani, aspetti naturalistici e storici*. Comune di San Giuliano Terme (PI), Felici Editore, Pisa, Italy, 2000, pp. 45-52.
37. Uncini Manganelli, R.E.; Camangi, F.; Tomei, P.E.; Oggiano L. L'uso delle erbe nella tradizione rurale della Toscana. ARSIA, Regione Toscana, Italy, 2002.
38. Perno, L.; Corsi, G.; Miraldi, E. Aspetti etnobotanici nel territorio di Rio nell'Elba. *Atti Soc. Tosc. Sci. Nat., Memorie, Serie B*, **1997**, *104,* 43-51.
39. Mattalia, G.; Sõukand, R.; Corvo, P.; Pieroni, A. Scholarly vs. traditional knowledge: Effects of sacred natural sites on ethnobotanical practices in Tuscany, Central Italy. *Hum. Ecol.* **2019**, *47*(5), 653-667.
40. Picci, G.; Pieroni, A. Atlante dei prodotti tipici: Le erbe. AGRA Eds.; Roma, Italy, 2005.
41. Mearelli, F.; Tardelli, C. Maremma Mediterranea. Erboristeria domani 1995, 7-8, 45-57.
42. Bonelli, G.; De Bellis, A.; Nanni G. Le insalate di campo raccolte sull'Amiata in Toscana. Effigi Eds.; Arcidosso, Grosseto, Italy, 2016.
43. Pieroni, A. Evaluation of the cultural significance of wild food botanicals traditionally consumed in Northwestern Tuscany, Italy. *J. Ethnobiol.* **2001**, *21*, 89-104.
44. Corsi, G.; Pagni, A.M. Piante selvatiche di uso alimentare in Toscana. Pacini Editore, Pisa, Italy, 2008.
45. Maccioni, S. I manoscritti del Museo Botanico Pisano, "Flora Economica della Provincia Pisana I" di Vincenzo Carmignani 1779-1859. *Atti Soc. Tosc. Sci. Nat., Memorie, Serie D,* **2015**, *122,* 5-9.
46. Camangi, F.; Stefani, A. Etnobotanica: flora spontanea e coltivata nella medicina popolare dell'Elba e dell'Arcipelago Toscano. Collana "Quaderni del Parco", Parco Nazionale Arcipelago Toscano, Bandecchi & Vivaldi, Pontedera, Pisa, Italy, 2016.
47. Camangi, F.; Tomei, T.E. Tradizioni etnofarmacobotaniche nella provincia di Livorno: il territorio della Valle Benedetta. *Inf. Bot. Ital.* **2003**, *35,* 41-54.
48. Uncini Manganelli, R.E.; Tomei, P.E. Indagini farmaco-botaniche in Garfagnana (Lucca): il versante appenninico). *Atti Soc. Tosc. Sci. Nat., Memorie, Serie D,* **1995**, *102,* 3-18.
49. Mambrini M.; Vicarelli G.B. Piante officinali dell'Amiata. Usi e tradizioni popolari. Cooperativa Agricola Forestale dei Comuni Amiatini, Castell'Azara Grosseto, Italy, 1983.

Italy

ALL REGIONS

1. Guarrera, P.M. Usi e tradizioni della flora italiana: medicina popolare ed etnobotanica. Aracne Editore, Roma, Italy, 2006.

MORE REGIONS

2. Ghirardini, M.P.; Carli, M.; Del Vecchio, N.; Rovati, A.; Cova, O.; Valigi, F.; Agnetti, G.; Macconi, M.; Adamo, D.; Traina, M.; et al. The importance of a taste. A comparative study on wild food plant consumption in twenty-one local communities in Italy. *J. Ethnobiol. Ethnomed.* **2007**, *3*, 22.
3. Idolo, M.; Motti, R.; Mazzoleni, S. Ethnobotanical and phytomedicinal knowledge in a long-history protected area the Abruzzo, Lazio and Molise National Park (Italian Apennines). *J. Ethnopharmacol.* **2010**, *127*, 379–395.

BASILICATA (BAS)

4. Giusti, M.E.; Nebel, S.; Pieroni, A. Erbe e percezione del sapore tra gli arbëreshë del Vulture in Lucania. *La Ricerca Folklorica* **2002**, *45*, 29-41.
5. Guarrera, P.M.; Salerno, G.; Caneva, G. Food, flavouring and feed plant traditions in the Tyrrhenian sector of Basilicata, Italy. *J. Ethnobiol. Ethnom.* **2006***, 2,* 37.
6. Pieroni, A.; Nebel, S.; Santoro, R.F.; Heinrich, M. Food for two seasons: Culinary uses of non-cultivated local vegetables and mushrooms in a south Italian village*. Int. J. Food Sci. Nut.* **2005***, 56*(4)*,* 245–272.
7. Sansanelli, S.; Ferri, M.; Salinitro, M.; Tassoni, A. Ethnobotanical survey of wild food plants traditionally collected and consumed in the Middle Agri Valley (Basilicata region, southern Italy). *J. Ethnobiol. Ethnomed.* **2017**, *13,* 50.

CALABRIA (CAL)

8. Mattalia, G.; Soukand, R.; Corvo, P.; Pieroni, A. Blended divergences: local food and medicinal plant uses among Arberesce, Occitans, and autochthonous Calabrians living in Calabria, Southern Italy. *Plant Biosyst.* **2020**, *154*(5), 615–626.
9. Nebel, S.; Heinrich, M. The Use of Wild Edible Plants in the Graecanic Area in Calabria, Southern Italy. In *Ethnobotany in the New Europe: People, Health and Wild Plant Resources*; Pardo-de-santayana, M., Pieroni, A., Puri, R.K., Eds.; Berghahn Books: Oxford, NY, USA, 2010; pp. 172–188.
10. Nebel, S.; Pieroni, A.; Heinrich, M. Ta chòrta: Wild edible greens used in the Graecanic area in Calabria, Southern Italy. *Appetite*, **2006**, *47*(3), 333–342.
11. Passalacqua, N.G.; De Fine, G.; Guarrera, P.M. Contribution to the knowledge of the veterinary science and of the ethnobotany in Calabria region (Southern Italy). *J. Ethnobiol. Ethnomed.* **2006**, *2*, 52.

CAMPANIA (CAM)

12. De Natale, A.; Pezzatti, G.B.; Pollio, A. Extending the temporal context of ethnobotanical databases: The case study of the Campania region (southern Italy). *J. Ethnobiol. Ethnomed.* **2009**, *5*, 7.
13. Di Novella, R.; Di Novella, N.; De Martino, L.; Mancini, E.; De Feo, V. Traditional plant use in the National Park of Cilento and Vallo di Diano, Campania, southern, Italy. *Journal of Ethnopharmacology,* **2013***, 145*(1), 328–342.
14. Guarino, C.; De Simone, L.; Santoro, S. Ethnobotanical study of the Sannio area, Campania, Southern Italy. *Ethnobot. Res. App.* **2008**, *6,* 255–317.
15. Mautone, M.; De Martino, L.; De Feo, V. Ethnobotanical research in Cava de' Tirreni area, Southern Italy. *J. Ethnobiol. Ethnomed.* **2019**, *15*, 50.
16. Motti, R.; Antignani, V.; Idolo, M. Traditional plant use in the Phlegraean Fields Regional Park (Campania, Southern Italy). *Hum. Ecol.* **2009***, 37*(6), 775–782.
17. Motti, R.; Bonanomi, G.; Lanzotti, V.; Sacchi, R. The contribution of wild edible plants to the mediterranean diet: an ethnobotanical case study along the coast of Campania (Southern Italy). *Econ. Bot.* **2020**, *74*(3), 249–272.
18. Salerno, G.; Guarrera, P.M. Ricerche etnobotaniche nel Parco Nazionale del Cilento e Vallo di Diano: il territorio di Castel San Lorenzo (Campania, Salerno). *Inf. Bot. Ital.* **2008***, 40*(2), 165–181.
19. Savo, V.; Salomone, F.; Bartoli, F.; Caneva, G. When the local cuisine still incorporates wild food plants: the unknown traditions of the Monti Picentini Regional Park (Southern Italy). *Econ. Bot.* **2019***, 73*(1), 28–46.

20. Scherrer, A.M.; Motti, R.; Weckerle, C.S. Traditional plant use in the areas of Monte Vesole and Ascea, Cilento National Park (Campania, Southern Italy). *J. Ethnopharmacol.* **2005***, 97*(1), 129–143.

EMILIA-ROMAGNA (EMI)

21. Sansanelli, S.; Tassoni, A. Wild food plants traditionally consumed in the area of Bologna (Emilia Romagna region, Italy). *J. Ethnobiol. Ethnomed.* **2014**, *10*, 69.

LIGURIA (LIG)

22. Cornara, L.; La Rocca, A.; Marsili, S.; Mariotti, M.G. Traditional uses of plants in the Eastern Riviera (Liguria, Italy). *J. Ethnopharmacol.* **2009**, *125*, 16–30.
23. Cornara, L.; La Rocca, A.; Terrizzano, L.; Dente, F.; Mariotti, M.G. Ethnobotanical and phytomedical knowledge in the North-Western Ligurian Alps. *J. Ethnopharmacol.* **2014***, 155*(1), 463–484.
24. Maccioni, S.; Monti, G.; Flamini, G.; Cioni, P.L.; Morelli, I.; Guazzi, E. Ricerche etnobotaniche in Liguria. La val Lerrone e la bassa valle Arroscia. *Atti Soc. Tosc. Sci. Nat., Memorie, Serie B*, **2004**, *111*, 129-134.

LOMBARDY (LOM)

25. Vitalini, S.; Iriti, M.; Puricelli, C.; Ciuchi, D.; Segale, A.; Fico, G. Traditional knowledge on medicinal and food plants used in Val San Giacomo (Sondrio, Italy) — An alpine ethnobotanical study. *J. Ethnopharmacol.* **2013***, 145*(2), 517–529.
26. Vitalini, S.; Puricelli, C.; Mikerezi, I.; Iriti, M. Plants, people and traditions: ethnobotanical survey in the Lombard Stelvio National Park and neighbouring areas (Central Alps, Italy). *J. Ethnopharmacol.* **2015***, 173,* 435–458.

THE MARCHES (MAR)

27. Lucchetti, L.; Zitti, S.; Taffetani, F. Ethnobotanical uses in the Ancona district (Marche region, Central Italy). *J. Ethnobiol. Ethnomed.* **2019**, *15,* 9.
28.

MOLISE (MOL)

29. Di Tizio, A.; Łuczaj, Ł.J.; Quave, C.L.; Redžić, S.; Pieroni, A. Traditional food and herbal uses of wild plants in the ancient South-Slavic diaspora of Mundimitar/Montemitro (Southern Italy). *J. Ethnobiol. Ethnomed.* **2012**, *8,* 21.
30. Menale, B.; Amato, G.; Di Prisco, C.; Muoio, R. Traditional uses of plants in north-western Molise (Central Italy). *Delpinoa* **2006**, *48,* 29–36.

PIEDMONT (PIE)

31. Pieroni, A.; Giusti, M.E. Alpine ethnobotany in Italy: traditional knowledge of gastronomic and medicinal plants among the Occitans of the upper Varaita valley, Piedmont. *J. Ethnobiol. Ethnomed.* **2009**, *5,* 32.

APULIA (PUG)

32. Biscotti, N.; Bonsanto, D.; Del Viscio, G. The traditional food use of wild vegetables in Apulia (Italy) in the light of Italian ethnobotanical literature. *Ital. Bot.* **2018**, *5,* 1–24.
33. Biscotti, N.; Del Viscio, G.; Bonsanto, D. Indagine etnobotanica sull'uso alimentare tradizionale di piante selvatiche in un comprensorio montano della regione Puglia

(Subappennino Dauno, provincia di Foggia). *Atti Soc. Tosc. Sci. Nat., Memorie, Serie B*, **2018**, *125*, 17-29.
34. Biscotti, N.; Pieroni, A. The hidden Mediterranean diet: wild vegetables traditionally gathered and consumed in the Gargano area, Apulia, SE Italy. *Acta Soc. Bot. Pol.* **2015**, *84*(3), 327–338.
35. Leporatti, M.L.; Guarrera, P.M. Ethnobotanical remarks in Capitanata and Salento areas (Puglia, southern Italy). *Etnobiología* **2007**, *5*(1), 51-64.
36. Pieroni, A.; Cattero, V. Wild vegetables do not lie: Comparative gastronomic ethnobotany and ethnolinguistics on the Greek traces of the Mediterranean Diet of southeastern Italy. *Acta Bot. Bras.* **2019**, *33*(2), 198–211.

SARDINIA (SAR)

37. Camarda, I.; Carta, L.; Vacca, G.; Brunu, A. Les plantes alimentaires de la Sardaigne: un patrimoine ethnobotanique et culturel d'ancienne origine. *Flora Mediter.* ***2017**, 27*, 77–90.
38. Lancioni, M.C.; Ballero, M.; Mura, L.; Maxia, A. Usi alimentari e terapeutici nella tradizione popolare del Goceano (Sardegna centrale). *Atti Soc. Tosc. Sci. Nat., Memorie, Serie B,* **2007**, *114,* 45–56.
39. Mattalia, G.; Soukand, R.; Corvo, P.; Pieroni, A. Wild food thistle gathering and pastoralism: an inextricable link in the biocultural landscape of Barbagia, central Sardinia (Italy). *Sustainability* **2020**, *12*(12), 5105.
40. Signorini, M.A.; Piredda, M.; Bruschi, P. Plants and traditional knowledge: An ethnobotanical investigation on Monte Ortobene (Nuoro, Sardinia). *J. Ethnobiol. Ethnomed.* **2009**, *5*, 6.

SICILY (SIC)

41. Cucinotta, F.; Pieroni, A. "If you want to get married, you have to collect virdura": the vanishing custom of gathering and cooking wild food plants on Vulcano, Aeolian Islands, Sicily. *Food Cult. Soc.* **2018**, *21*(4), 539-567.
42. Lentini, F.; Venza, F. Wild food plants of popular use in Sicily. *J. Ethnobiol. Ethnomed.* **2007**, *3,* 15.
43. Licata, M.; Tuttolomondo, T.; Leto, C.; Virga, G.; Bonsangue, G.; Cammalleri, I.; Gennaro, M.C.; La Bella, S. A survey of wild plant species for food use in Sicily (Italy)—results of a 3-year study in four Regional Parks. *J. Ethnobiol. Ethnomed.* **2016**, 12, 12.
44. Pasta, S.; La Rosa, A.; Garfì, G.; Marcenò, C.; Gristina, A.S.; Carimi, F.; Guarino, R. An updated checklist of the sicilian native edible plants: preserving the traditional ecological knowledge of landscapes. *Front. Plant Sci.* **2020**, *11,* 388.

UMBRIA (UMB)

45. Ranfa, A.; Bodesmo, M. An ethnobotanical investigation of traditional knowledge and uses of edible wild plants in the Umbria Region, Central Italy. *J. Appl. Bot. Food Qual.* **2017**, *90,* 246–258.
46. Ranfa, A.; Maurizi, A.; Romano, B.; Bodesmo, M. The importance of traditional uses and nutraceutical aspects of some edible wild plants in human nutrition: the case of Umbria (central Italy). *Plant Biosyst.* **2014**, *148*(2), 297–306.

VENETO (VEN)

47. Zuin, M.C. (2010). Piante alimurgiche del Veneto. Riconoscerle, coltivarle e gustarle. Regione Veneto, Venezia.

FRIULI-VENEZIA GIULIA (FRI)

48. Dreon, A.L.; Paoletti, M.G. The wild food (plants and insects) in Western Friuli local knowledge (Friuli-Venezia Giulia, North Eastern Italy). *Contrib. Nat. Hist.* **2009**, *12(1)*, 461-488.

Europe

MORE COUNTRIES

1. Pardo-de-Santayana, M.; Tardío, J.; Blanco, E.; Carvalho, A.M.; Lastra, J.J.; San Miguel, E.; Morales, R. Traditional knowledge of wild edible plants used in the northwest of the Iberian Peninsula (Spain and Portugal): A comparative study. *J. Ethnobiol. Ethnomed.* **2007**, *3,* 27.
2. Sõukand, R.; Pieroni, A. The importance of a border: Medical, veterinary, and wild food ethnobotany of the Hutsuls living on the Romanian and Ukrainian sides of Bukovina. *J. Ethnopharmacol.* **2016**, *185,* 17–40.
3. Sõukand, R.; Pieroni, A.; Biró, M.; Dénes, A.; Dogan, Y.; Hajdari, A.; Kallea, R.; Reade, B.; Mustafa, B.; Nedelcheva, A.; et al. An ethnobotanical perspective on traditional fermented plant foods and beverages in Eastern Europe. *J. Ethnopharmacol.* **2015**, *170*, 284–296.
4. Sõukand, R.; Quave, C.L.; Pieroni, A.; Pardo-De-Santayana, M.; Tardío, J.; Kalle, R.; Łuczaj, Ł.; Svanberg, I.; Kolosova, V.; Aceituno-Mata, L.; et al. Plants used for making recreational tea in Europe: A review based on specific research sites. *J. Ethnobiol. Ethnomed.* **2013**, *9*, 58.

ALBANIA (AL)

5. Pieroni, A. Local plant resources in the ethnobotany of Theth, a village in the Northern Albanian Alps. *Genet. Resour. Crop Evol.* **2008**, *55*(8), 1197–1214.
6. Pieroni, A.; Cianfaglione, K.; Nedelcheva, A.; Hajdari, A.; Mustafa, B.; Quave, C.L. Resilience at the border: Traditional botanical knowledge among Macedonians and Albanians living in Gollobordo, Eastern Albania. *J. Ethnobiol. Ethnomed.* **2014***, 10*, 31.
7. Pieroni, A.; Ibraliu, A.; Abbasi, A.M.; Papajani-Toska, V. An ethnobotanical study among Albanians and Aromanians living in the Rraicë and Mokra areas of Eastern Albania. *Genet. Resour. Crop Evol.* **2015**, *62*(4), 477–500.
8. Pieroni, A.; Nedelcheva, A.; Hajdari, A.; Mustafa, B.; Scaltriti, B.; Cianfaglione, K.; Quave, C.L. Local knowledge on plants and domestic remedies in the mountain villages of Peshkopia (Eastern Albania). *J. Mt. Sci.* **2014**, *11*(1), 180–193.
9. Pieroni, A.; Sõukand, R. The disappearing wild food and medicinal plant knowledge in a few mountain villages of North-Eastern Albania. *J. App. Bot. Food Qual.* **2007**, *90,* 58–67.
10. Quave, C.L.; Pieroni, A. Fermented foods for food security and food sovereignty in the Balkans: A case study of the gorani people of Northeastern Albania. *Journal of Ethnobiology,* **2014**, *34*(1), 28–43.

ARMENIA (AR)

11. Nanagulyan, S.: Zakaryan, N.; Kartashyan, N.; Piwowarczyk, R.; Łuczaj, Ł. Wild plants and fungi sold in the markets of Yerevan (Armenia). *J. Ethnobiol. Ethnomed.* **2020**, *16*, 26.
12. Pieroni, A.; Hovsepyan, R.; Manduzai, A.K.; Sõukand, R. Wild food plants traditionally gathered in central Armenia: archaic ingredients or future sustainable foods? *Environ. Dev. Sustain.* **2020**, *23*(2), 2358-2381.

AUSTRIA (AT)

13. Schunko, C.; Vogl, C.R. Organic farmers use of wild food plants and fungi in a hilly area in Styria (Austria). *J. Ethnobiol. Ethnomed.* **2010**, *6*, 17.

BELARUS (BY)

14. Łuczaj, Ł.; Köhler, P.; Piroznikow, E.; Graniszewska, M.; Pieroni, A.; Gervasi, T. Wild edible plants of Belarus: From Rostafiński's questionnaire of 1883 to the present. *J. Ethnobiol. Ethnomed.* **2013**, *9,* 21.
15. Sõukand, R.; Hrynevich, Y.; Vasilyeva, I.; Prakofjewa, J.; Vnukovich, Y.; Paciupa, J.; Hlushko, A.; Knureva, Y.; Litvinava, Y.; Vyskvarka, S.; et al. Multi-functionality of the few: Current and past uses of wild plants for food and healing in Liubań region, Belarus. *J. Ethnobiol. Ethnomed.* **2017**, *13, 10*.

BOSNIA-HERZEGOVINA (BA)DOLINA

16. Łuczaj, Ł.; Dolina, K. A hundred years of change in wild vegetable use in southern Herzegovina. *J. Ethnopharmacol.* **2015**, *166,* 297–304.
17. Redžić, S. Wild edible plants and their traditional use in the human nutrition in Bosnia-Herzegovina. *Ecol. Food Nutr.* **2006**, *45*(3), 189–232.
18. Redžić, S. Use of wild and semi-wild edible plants in nutrition and survival of people in 1430 days of siege of Sarajevo during the war in Bosnia and Herzegovina (1992-1995). *Col. Antropol.* **2010**, *34*(2), 551–570.
19. Redžić, S.; Ferrier, J. The use of wild plants for human nutrition during a war: eastern Bosnia (Western Balkans). In *Ethnobotany and biocultural diversities in the Balkans*; Pieroni A., Quave C., Eds.; Springer: New York, NY, USA. 2014; pp. 149-182.

BULGARIA (BG)

20. Ivanova, T.A.; Bosseva, Y.Z.; Ganeva-Raycheva, V.G.; Dimitrova, D. Ethnobotanical knowledge on edible plants used in zelnik pastries from Haskovo province (Southeast Bulgaria). *Phytol. Balcan.* **2018**, *24*(3), 389-395.
21. Nedelcheva, A. An ethnobotanical study of wild edible plants in Bulgaria. *EurAsia. J. BioSci.* **2013**, *7*, 77–94.

CROATIA (HR)

22. Dolina, K.; Jug-Dujaković, M.; Łuczaj, Ł.; Vitasović-Kosić, I. A century of changes in wild food plant use in coastal Croatia: The example of Krk and Poljica. *Acta Soc. Bot. Pol.* **2016***, 85*(3), 1–22.
23. Dolina, K.; Łuczaj, Ł. Wild food plants used on the Dubrovnik coast (South-Eastern Croatia). *Acta Soc. Bot. Pol.* **2014***, 83*(3), 175–181.
24. Łuczaj, Ł.; Fressel, N.; Perković, S. Wild food plants used in the villages of the Lake Vrana Nature Park (northern Dalmatia, Croatia). *Acta Soc. Bot. Pol.* **2013**, *82*(4), 275–281.
25. Łuczaj, Ł.; Jug-Dujaković, M.; Dolina, K.; Jeričević, M.; Vitasović-Kosić, I. The ethnobotany and biogeography of wild vegetables in the Adriatic islands. *J. Ethnobiol. Ethnomed.* **2019**, *15*, 18.
26. Łuczaj, Ł.; Jug-Dujaković, M.; Dolina, K.; Vitasović-Kosić, I. Plants in alcoholic beverages on the Croatian islands, with special reference to rakija travarica. *J. Ethnobiol. Ethnomed.* **2019**, *15*, 51.
27. Łuczaj, Ł.; Zovko Končić, M.; Miličević, T.; Dolina, K.; Pandža, M. Wild vegetable mixes sold in the markets of Dalmatia (southern Croatia). *J. Ethnobiol. Ethnomed.* **2013**, *9,* 2.
28. Varga, F.; Šolić, I.; Dujaković, M.J.; Łuczaj, Ł.; Grdiša, M. The first contribution to the ethnobotany of inland Dalmatia: Medicinal and wild food plants of the Knin area, Croatia.

Acta Soc. Bot. Pol. **2019**, *88*(2), 1–20.
29. Vitasović Kosić, I.; Juračak, J.; Łuczaj, Ł. Using Ellenberg-Pignatti values to estimate habitat preferences of wild food and medicinal plants: An example from northeastern Istria (Croatia). *J. Ethnobiol. Ethnomed.* **2017**, *13,* 31.

CYPRUS (CY)
30. Della, A.; Paraskeva-Hadjichambi, D.; Hadjichambis, A.C. An ethnobotanical survey of wild edible plants of Paphos and Larnaca countryside of Cyprus. *J. Ethnobiol. Ethnomed.* **2006**, *2,* 34.

CZECH REPUBLIC (CZ)
31. Pawera, L.; Łuczaj, Ł.; Pieroni, A.; Polesny, Z. Traditional plant knowledge in the white carpathians: ethnobotany of wild food plants and crop wild relatives in the Czech Republic. *Hum. Ecol.* **2017**, *45*(5), 655–671.
32. Simkova, K.; Polesny, Z. Ethnobotanical review of wild edible plants used in the Czech Republic. *J. App. Bot. Food Qual.* **2015**, *88*, 49–67.

ESTONIA (EE)
33. Kalle, R.; Sõukand, R. Historical ethnobotanical review of wild edible plants of Estonia (1770s-1960s). *Acta Soc. Bot. Pol.* **2012**, *81*(4), 271–281.
34. Kalle, R.; Sõukand, R. Wild plants eaten in childhood: a retrospective of Estonia in the 1970s – 1990s. *Bot. J. Linn. Soc.* **2013**, *172*, 239–253.
35. Kalle, R.; Sõukand, R. Current and remembered past uses of wild food plants in saaremaa, estonia: Changes in the context of unlearning debt. *Econ. Bot.* **2016**, *70*(3), 235–253.
36. Kalle, R.; Sõukand, R.; Pieroni, A. Devil is in the details: Use of wild food plants in historical Võromaa and Setomaa, present-day Estonia. *Foods* **2020**, *9*(5).
37. Sõukand, R.; Kalle, R. Perceiving the biodiversity of food at chest-height: Use of the fleshy fruits of wild trees and shrubs in Saaremaa, Estonia. *Hum. Ecol.* **2016**, *44*(2), 265–272.

GEORGIA (GE)
38. Bussmann, R.W.; Paniagua Zambrana, N.J.; Sikharulidze, S.; Kikvidze, Z.; Darchidze, M.; Manvelidze, Z.; Ekhvaia, J.; Kikodze, D.; Tchelidze, D.; Khutsishvili, M. et al. From the sea to the mountains - plant use in Ajara, Samegrelo and Kvemo Svaneti, Sakartvelo (Republic of Georgia), Caucasus. *Ethnob. Res. Appl.* **2020**, *20,* 1–34.

GREECE (EL)
39. Pieroni, A.; Cattero, V. Wild vegetables do not lie: Comparative gastronomic ethnobotany and ethnolinguistics on the Greek traces of the Mediterranean Diet of southeastern Italy. *Acta Bot. Bras.* **2019**, *33*(2), 198–211.

KOSOVO (XK)
40. Mustafa, B.; Hajdari, A.; Pulaj, B.; Quave, C.L.; Pieroni, A. Medical and food ethnobotany among Albanians and Serbs living in the Shtërpcë/Štrpce area, South Kosovo. *J. Herb. Med.* **2020**, *22*, 100344.
41. Pieroni, A.; Sõukand, R.; Quave, C.L.; Hajdari, A.; Mustafa, B. Traditional food uses of wild plants among the Gorani of South Kosovo. *Appetite,* **2017**, *108,* 83–92.

MACEDONIA (MK)

42. Nedelcheva, A.; Pieroni, A.; Dogan, Y. Folk food and medicinal botanical knowledge among the last remaining Yörüks of the Balkans. *Acta Soc. Bot. Pol.* **2017**, *86*(2), 1–21.
43. Pieroni, A.; Rexhepi, B.; Nedelcheva, A.; Hajdari, A.; Mustafa, B.; Kolosova, V.; Cianfaglione, K.; Quave, C.L. One century later: The folk botanical knowledge of the last remaining Albanians of the upper Reka Valley, Mount Korab, Western Macedonia. *J. Ethnobiol. Ethnomed.* **2013**, *9*, 22.
44. Rexhepi, B.; Bajrami, A.; Mustafa, B. Ethnobotanical study of wild edible plants in Pelagonia Region (Southwestern Macedonia). *Int. J. Adv. Sci. Eng. Technol.* **2018**, 6, 57-61.

NETHERLANDS (NL)

45. Vorstenbosch, T.; de Zwarte, I.; Duistermaat, L.; Van Andel, T. Famine food of vegetal origin consumed in the Netherlands during World War II. *J. Ethnobiol. Ethnomed.* **2017**, *13,* 63.

NORWAY (NO)

46. Teixidor-Toneu, I.; Kjesrud, K.; Bjerke, E.; Parekh, K.; Kool, A. From the "Norwegian Flora" (Eighteenth Century) to "Plants and Tradition" (Twentieth Century): 200 years of Norwegian knowledge about wild plants. *Econ. Bot.* **2020***, 10*, 1–13.

POLAND (PL)

47. Kasper-Pakosz, R.; Pietras, M.; Łuczaj, Ł. Wild and native plants and mushrooms sold in the open-air markets of south-eastern Poland. *J. Ethnobiol. Ethnomed.* **2016**, *12*, 45.
48. Łuczaj, L. Archival data on wild food plants used in Poland in 1948. *J. Ethnobiol. Ethnomed.* **2008**, *4*, 4.
49. Łuczaj, Ł. Changes in the utilization of wild green vegetables in Poland since the 19th century: A comparison of four ethnobotanical surveys. *J. Ethnopharmacol.* **2010**, *128*(2), 395–404.
50. Łuczaj, Ł.; Szymański, W.M. Wild vascular plants gathered for consumption in the Polish countryside: A review. *J. Ethnobiol. Ethnomed.* **2007**, *3,* 17.

PORTUGAL (PT)

51. Vinagre, C.; Vinagre, S.; Carrilho, E.; García, D.; Vázquez F.M., Pinto Gomes, C. Ethnobotanical study in the protected landscape "Serra de Montejunto" (Portugal). *J. Med. Plants* **2017**, *5*(1), 110-124.

ROMANIA (RO)

52. Pieroni, A.; Nedelcheva, A.; Dogan, Y. Local knowledge of medicinal plants and wild food plants among Tatars and Romanians in Dobruja (South-East Romania). *Genet. Resour. Crop Evol.* **2015**, *62*(4), 605–620.
53. Vári, Á.; Arany, I.; Kalóczkai, Á.; Kelemen, K.; Papp, J.; Czúcz, B. Berries, greens, and medicinal herbs - Mapping and assessing wild plants as an ecosystem service in Transylvania (Romania). *J. Ethnobiol. Ethnomed.* **2020**, *16, 13*.

RUSSIA (RU)

54. Kolosova, V.; Belichenko, O.; Rodionova, A.; Melnikov, D.; Sõukand, R. Foraging in boreal forest: Wild food plants of the republic of Karelia, NW Russia. *Foods* **2020**, 9(8).

SLOVAKIA (SK)

55. Končeková, L.; Halmová, D.; Fehér, A. Edible wild plants growing in adjacent spontaneous vegetation of energy plantations in southwest Slovakia. *Slovak J. Food Sci.* **2020**, *14*, 1–7.
56. Łuczaj, Ł. Ethnobotanical review of wild edible plants of Slovakia. *Acta Soc. Bot. Pol.* **2012**,

81(4), 245–255.

SPAIN (ES)

57. Alarcón, R.; Pardo-De-Santayana, M.; Priestley, C.; Morales, R.; Heinrich, M. Medicinal and local food plants in the south of Alava (Basque Country, Spain). *J. Ethnopharmacol.* **2015**, *176,* 207–224.
58. Blanco-Salas, J.; Gutiérrez-García, L.; Labrador-Moreno, J.; Ruiz-Téllez, T. Wild plants potentially used in human food in the protected area “Sierra Grande de Hornachos” of extremadura (Spain). *Sustainability* **2019**, *11*(2).
59. González, J.A.; García-Barriuso, M.; Amich, F. The consumption of wild and semi-domesticated edible plants in the Arribes del Duero (Salamanca-Zamora, Spain): An analysis of traditional knowledge. *Genet. Resour. Crop Evol.* **2011**, *58*(7), 991–1006.
60. Gutiérrez-García, L.; Labrador-Moreno, J.; Blanco-Salas, J.; Monago-Lozano, F.J.; Ruiz-Téllez, T. Food identities, biocultural knowledge and gender differences in the protected area “sierra grande de hornachos” (Extremadura, spain). *Int. J. Environ. Res. Public Health* **2020**, *17*(7).
61. Menendez-Baceta, G.; Aceituno-Mata, L.; Tardío, J.; Reyes-García, V.; Pardo-de-Santayana, M. Wild edible plants traditionally gathered in Gorbeialdea (Biscay, Basque Country). *Genet. Resour. Crop Evol.* **2012**, *59*(7), 1329–1347.
62. Parada, M.; Carrió, E.; Vallès, J. Ethnobotany of food plants in the alt empordà region (catalonia, iberian peninsula). *J. App. Bot. Food Qual.* **2011**, *84*(1), 11–25.
63. Pardo-De-Santayana, M.; Tardío, J.; Morales, R. The gathering and consumption of wild edible plants in the Campoo (Cantabria, Spain). *Int. J. Food Sci. Nut.* **2005**, *56*(7), 529–542.
64. Rigat, M.; Gras, A.; D’Ambrosio, U.; Garnatje, T.; Parada, M.; Vallès, J. Wild food plants and minor crops in the Ripollès district (Catalonia, Iberian Peninsula): Potentialities for developing a local production, consumption and exchange program. J. Ethnobiol. Ethnomed. **2016**, 12, 49.
65. Talavera Roma, M.; Ninot Sugrañes, J.M.; Solé Cantero, A. Organic wild species crops: A tool to recover traditional knowledge, and to enhance biodiversity, food security and sustainable rural development. *Acta Fytotec. Zootec.* **2015**, *18*, 62–64.
66. Tardío, J.; Pardo-De-Santayana, M.; Morales, R. Ethobotanical review of wild edible plants in Spain. *Bot. J. Linn. Soc.* **2006**, *152,* 27-71.

SWITZERLAND (CH)

67. Abbet, C.; Mayor, R.; Roguet, D.; Spichiger, R.; Hamburger, M.; Potterat, O. Ethnobotanical survey on wild alpine food plants in Lower and Central Valais (Switzerland). *J. Ethnopharmacol.* **2014**, *151*(1), 624–634.

UCRAINA (UA)

68. Pieroni, A.; Sõukand, R. Are borders more important than geographical distance? The wild food ethnobotany of the boykos and its overlap with that of the Bukovinian Hutsuls in Western Ukraine. *J. Ethnobiol.* **2017**, *37*(2), 326–345.
69. Pieroni, A.; Sõukand, R. Forest as stronghold of local ecological practice: Currently used wild food plants in Polesia, Northern Ukraine. *Econ. Bot.* **2018**, *72*(3), 311–331.
70. Sõukand, R.; Stryamets, N.; Fontefrancesco, M.F.; Pieroni, A. The importance of tolerating interstices: Babushka markets in Ukraine and Eastern Europe and their role in maintaining local food knowledge and diversity. *Heliyon* **2020**, *6*, E03222.
